# On the Nature of the Partial Covalent Bond between Noble Gas Elements and Noble Metal Atoms

**DOI:** 10.3390/molecules28073253

**Published:** 2023-04-05

**Authors:** Ranita Pal, Pratim Kumar Chattaraj

**Affiliations:** 1 Advanced Technology Development Centre, Indian Institute of Technology, Kharagpur 721302, India; 2 Department of Chemistry, Indian Institute of Technology, Kharagpur 721302, India

**Keywords:** chemical bonding, noble gas, noble metals, partial covalent bond, conceptual DFT

## Abstract

This article provides a discussion on the nature of bonding between noble gases (Ng) and noble metals (M) from a quantum chemical perspective by investigating compounds such as NgMY (Y=CN, O, NO_3_, SO_4_, CO_3_), [NgM−(bipy)]+, NgMCCH, and MCCNgH complexes, where M=Cu, Ag, Au and Ng=Kr−Rn, with some complexes containing the lighter noble gas atoms as well. Despite having very low chemical reactivity, noble gases have been observed to form weak bonds with noble metals such as copper, gold, and silver. In this study, we explore the factors that contribute to this unusual bonding behavior, including the electronic structure of the atoms involved and the geometric configuration of the concerned fragments. We also investigate the metastable nature of the resulting complexes by studying the energetics of their possible dissociation and internal isomerization channels. The noble gas-binding ability of the bare metal cyanides are higher than most of their bromide counterparts, with CuCN and AgCN showing higher affinity than their chloride analogues as well. In contrast, the oxides seem to have lower binding power than their corresponding halides. In the oxide and the bipyridyl complexes, the Ng-binding ability follows the order Au > Cu > Ag. The dissociation energies calculated, considering the zero-point energy correction for possible dissociation channels, increase as we move down the noble gas group. The bond between the noble gases and the noble metals in the complexes are found to have comparable weightage of orbital and electrostatic interactions, suggestive of a partial covalent nature. The same is validated from the topological analysis of electron density.

## 1. Introduction

Chemical bonding is a fundamental concept in chemistry that encompasses the interactions between atoms and the forces that hold them together. This phenomenon plays a crucial role in the formation of chemical compounds and the behavior of their constituent elements. Understanding the nature of chemical bonds is essential for predicting the stability, reactivity, and properties of chemical compounds, as well as for designing new materials with specific characteristics. We have studied ‘covalent’ and ‘ionic’ types of bonding in elementary chemistry, each of which are influenced by the electronic structure of the concerned fragments. But how true are the definitions of covalent and ionic bonds? In theory, there can exist a 100% ionic bond between two atoms, where one atom completely donates its valence electrons to the other atom, resulting in the formation of a completely ionic compound. However, in practice, no such bond can exist because all chemical bonds involve some degree of electron sharing as the orbitals of the cations and the anions may not be just spectators. In most cases, even compounds that are considered to have predominantly ionic bonding, e.g., ionic salts such as NaCl, have some degree of covalent character. Again, most ‘covalent’ bonds have some degree of ionic character, even if it is very small. This is because the electronegativities of the atoms in a bond are rarely exactly the same, leading to an unequal sharing of electrons. Additionally, other factors such as bond polarity and resonance can also affect the degree of covalency in a bond. Even in homonuclear molecules, the presence of ionic character is shown, and this is discussed later in the article. Basically, most bonds that exist can be called ‘partially covalent’, rather than ‘covalent’ or ‘ionic’.

Even noble gas (Ng) atoms, known for their high stability and low reactivity, are reported to form partial covalent bonds with other elements. Their inertness is justified by their high first ionization potentials (IPs) which decreases monotonically from He to Rn. The heavier Ngs can therefore have one electron knocked out of their outermost shell, making Ng compounds feasible. Kossel anticipated that the fluorides for Kr and Xe should be able to exist [1]. Antropoff asserted in 1924 that because Ng atoms may increase their valence up to eight, they belong to Group 18 [2]. Pauling predicted that xenon can bind with oxygen to generate xenic acid (H_4_XeO_6_), and that it “should form salts such as Ag_4_XeO_6_ and AgH_3_XeO_6_” [3]. This prediction was based on ionic radii. Pauling further predicted the occurrence of krypton and xenon hexafluorides (KrF_6_ and XeF_6_) and xenon octafluoride (XeF_8_) based on the observation that the radius ratio of oxygen and fluorine is 1.29. However, up until 1962, the several attempts to manufacture Ng molecules were unsuccessful. In 1962, during his experiments involving the oxidation of O_2_ by PtF_6_ producing O_2_^+^PtF_6_^−^ [4], Bartlett realized the similarity in the first IP of molecular oxygen with xenon, which might lead to a similar oxidation in the latter; this guided the synthesis of the first xenon compound, the orange-yellow colored XePtF_6_ [5,6,7]. 

The discovery of the first Ng compound shattered the long-held belief that noble gases do not form chemical bonds, opening up a new field of study called “Noble Gas Chemistry.” At first, Xe^+^PtF_6_^-^ was believed to be the formula for the first Ng compound, but later, X-ray powder diffraction images showed that XeF^+^Pt_2_F_11_^-^ existed instead [8]. Further, research revealed the mechanism of the formation of XeF^+^Pt_2_F_11_^-^ and other noble gas compounds, such as XeF_2_, XeF_4_, XeF_6_, XeOF_4_, XeO_3_, and KrF_2_, which were synthesized, characterized, and reported within a year of Bartlett’s discovery [9,10]. Xenon has been extensively researched among the noble gases due to its weakly bound electrons, and has many documented compounds in the literature [11,12,13,14,15,16]. Even the radioactive Ng atom, Rn, has been known to successfully form compounds [17]. Ar compounds were isolated in 2000 [18,19], and weak Ne complexes were identified in a low-temperature matrix [20,21,22,23]. Recently, it was discovered that even He can participate in chemical bonding at high pressures, forming a solid Na_2_He compound with an 8c-2e bonding structure [24]. Theoretical contributions to the study of noble gas compounds can be categorized into three primary groups: non-insertion (NgXY), insertion (XNgY), and cage complexes with Ng encapsulation.

In a coupled-cluster method (CCSD(T)) analysis in 1995, Pyykko [25] predicted the stability of two cationic gold compounds, NgAu^+^ and NgAuNg^+^. Three years later, these were also discovered experimentally [26]. The existence of considerable covalent character in the interaction of Au with heavy Ng atoms was also proposed [25,26]. However, this interaction between Ng and Au was claimed to be the result of polarization and long-range dispersion effects by Buckingham et al. [27]. A few years later, Gerry et al. identified the Ar and Kr analogues of NgAuCl using rotational spectroscopy [28], while Seidel et al. discovered the compound AuXe_4_^2+^ while trying to extract AuF [29]. These discoveries led to the exploration of more Ng-transition metal compounds, where Ng atoms played the role of weak ligands. Gerry et al. further investigated these compounds and characterized a series of NgMX (Ng=Ar, Kr, Xe; M=Cu, Ag, Au; X=F, Cl, Br) compounds spectroscopically [30,31,32,33,34], while theoretical studies focused on exploring their bonding nature and stability [35,36,37,38,39,40]. Recently, Wang et al. used matrix infrared spectroscopy to detect NeAuF [41]. The Ng-binding ability of MOH (M=Cu, Ag, Au) was also studied theoretically [42,43]. Additionally, Ghanty proposed the insertion of Ng atoms within M-F or M-OH (M=Cu, Ag, Au) [44,45].

In this review article, we will discuss the bonding and stability of a few of these Ng compounds that our group has worked on over the years, especially those where the Ng atom attempts to bind to a noble metal M (Cu, Ag, Au). The unusual bonding interaction between two low reactive elements has long captured our attention. Here we provide a brief discussion on the stability and bonding of the type of complexes they can form.

## 2. Computational Tools to Analyze the Bonding

The bonding nature of these types of compounds are typically analyzed using tools based on computational techniques, viz., Natural Bond Orbital (NBO), Quantum Theory of Atoms-in-Molecules (QTAIM), Electron Localization Function (ELF), and Energy Decomposition Analysis (EDA), among others.

The Natural Bond Orbital (NBO) [46] is based on the concept of localized chemical bonding, where orbitals are constructed for individual chemical bonds in a molecule. It uses the density matrix of a molecule to obtain information about the probability distribution of electrons in the molecule. The NBO analysis, along with the Wiberg bond index (WBI) [47], provides a detailed picture of the bonding pattern in a molecule, including the degree of covalency, polarization, and charge transfer. WBI is a quantitative measure of the degree of electron sharing between two atoms in a molecule and was developed by Professor Steven R. Wiberg. It is based on the molecular orbital theory, specifically, on the concept of natural atomic orbitals, and it is evaluated from the diagonal elements of the density matrix of the molecular orbitals of a molecule. In other words, it is based on the density of electrons shared between two atoms. It ranges from zero to the sum of the formal bond orders between two atoms. A WBI value of zero indicates that there is no electron sharing between the two atoms, while a value equal to the sum of the formal bond orders indicates complete electron sharing. A higher value of WBI indicates a higher degree of covalent character in the concerned bond.

In QTAIM, a zero-flux surface defines an atom to obtain its atomic energy, even while it is a part of a molecule, by assuming the atoms to be the fundamental units rather than the bonds [48,49,50]. Here, the bonding is described in terms of the topology of the electron density in the system by partitioning it through the steepest ascent trajectory or the gradient path (GP). ELF is also based on the same principle [51,52]. Despite being widely used by scientists globally, the QTAIM approach has been subject to reports questioning its accuracy and foundation, specifically in relation to QTAIM atomic charges [53,54,55,56]. There also exist some debates regarding the accuracy of the atomic charges used in the QTAIM approach and their ability to reproduce the dipole/quadrupole moment or the electrostatic potential of a molecule. Some reports indicate a “size dependency” in the QTAIM charges [57] and a wrong indication of the ionic bond in the CO molecule due to the charge on the C center being unexpectedly high [53]. According to Bader and Matta [58], the consideration of atomic polarizations is essential in charge transfer calculations to avoid inaccurate QTAIM charges, as a point-charge model fails to account for them. Additionally, they note that quantum mechanics cannot provide a unique definition of atomic charge [55] as it is not a physical observable. This observation contradicts the principles of an open system in physics [59]. However, Bader and Matta [58] argue that atomic charges can be described as a quantum expectation value for an open system and can therefore be used to calculate external field-induced polarization and permanent moments in molecules. However, they also caution that atomic charges defined outside the context of an open system have limited usefulness, as they cannot be used in quantum mechanical expressions for expectation values and are not related to measurable properties. Electron density (ED, *ρ*(*r*)), Laplacian of ED (▽^2^*ρ*(*r*)), local energy densities (kinetic *G*(*r*), and potential *V*(*r*)), and their sum (*H*(*r*)) calculated at the bond critical point (BCP) are some of the parameters used to describe the bonding. The conventional criterion for identifying the nature of a bond, which involves checking for the sign of ▽^2^*ρ*(*r*) at the BCP is often not enough for Ng compounds. This could be due to the weaker orbital involvement of Ng in bonding compared to other elements in the periodic table, or that the criterion tends to fail for heavier elements beyond the first row of the periodic table. The reason for this is that ▽^2^*ρ*(*r*) is calculated from the curvatures λ_1_, λ_2_, and λ_3_, of which the first two are negative while the final term is positive. For heavier elements, the positive contribution from the last term can often dominate, resulting in a positive ▽^2^*ρ*(*r*) value. In such cases, the *H*(*r*) can be a more useful descriptor whose negative value indicates the presence of covalent (or partial covalent) bonding depending on the magnitude, even when ▽^2^*ρ*(*r*) is positive. The full topological analysis of *H*(***r***) is reported as an effective mode to investigate the nature of the bonds occurring in Ng compounds in cases where they have examined its plotted shape, topology, and value along the AIM bond path, particularly in the vicinity of the BCP. This allows for a systematic classification of the concerned bonds using a range of numerical indices that are specific to each type of bond [60]. A few other interesting publications have been reported on the full bonding analysis in noble gas compounds [61,62,63].

Energy Decomposition Analysis (EDA) is another powerful computational tool used in the field of quantum chemistry to analyze the interaction between molecules [64,65]. It is used to decompose the total interaction energy between fragments of a molecular system into various energy components, which correspond to different types of interactions between them. These energy components can be used to analyze the nature and strength of the intermolecular interactions, thereby explaining the possible bonding therein. The components of the said energy are of attractive and repulsive types. The former constitutes electrostatic (Coulombic interaction between the EDs of the two fragments; *E*_elstat_), orbital (*E*_orb_), and dispersion (attractive van der Waals forces between the fragments which arise due to the fluctuations in the corresponding EDs; *E*_disp_) interaction energies, while the latter is essentially the Pauli repulsion interaction (*E*_Pauli_). To examine the bonding of the Ng compounds, we took into account both neutral and ionic fragmentation methods during the EDA computations. The neutral and ionic fragmentation schemes represent electron-shared covalent interaction and dominant electrostatic interaction, respectively. To identify the better partitioning scheme, we compared the Δ*E*_orb_ values of both. The partitioning scheme with a smaller attractive Δ*E*_orb_ value represents the bonding structure more accurately, as it causes less electronic charge reorganization to yield the electronic structure of the compound [66,67].

In our discussions, we have described the bonding in terms of %*covalent* and %*ionic* characters. These percentages are calculated as:

(1) $$\%covalent=\frac{E_{orb}}{E_{orb}+E_{elstat}}*100$$

(2) $$\%ionic=100-\left(\%covalent\right)=\frac{E_{elstat}}{E_{orb}+E_{elstat}}*100$$

It may be noted that these definitions are mostly qualitative in nature. In the complexes of Ng with metal cations, the term Δ*E*_orb_ obtained by the EDA scheme employed is dominated by the inductive interaction, which is “physical” in nature and does not necessarily demand the sharing of electrons that is typical of covalent bonds. Similarly, in the majority of the Ng complexes, the Δ*E*_elstat_ arises from the interaction between the frozen densities of the interacting fragments, which is different from what is commonly perceived as an ionic bond or an ionic contribution to the bond. For simple molecules in general, those definitions are expected to suffice. It is to be noted, however, that this simplified analysis is not perfectly applicable for bonds that are charge-shifting or eventually spin-polarized. For example, the F_2_ molecule is a well-known example of a charge-shifting bond. There are, of course, other ways to classify a chemical bond with detailed bonding analysis, particularly for important cases such as a charge-shifting bond. The chemical bond is not a physical observable, and there exists a high degree of arbitrariness during its analysis, especially when dealing with bonds with a high dispersion component, as is the case with noble gas bonds. Equations (1) and (2) are suggested in a qualitative sense, as they are capable of providing a rough idea about the extent of covalency in a typical bond.

We have performed the geometry optimizations, NBO and WBI, using Gaussian 09 [68], AIM analysis using Multiwfn [69], and EDA using the ADF 2013.01 package [70].

## 3. The General Bonding Picture

The notion of a conventional bond is not always well defined when referring to standard compounds with non-covalent interactions, such as traditional ionic compounds. In cases where two ions are attracted to each other by electrostatic forces, there is usually some amount of orbital overlap. Similarly, some amount of ionic character exists in traditional covalent compounds. We have provided a description of the bonding picture in some commonly known diatomic ‘covalent’ and ‘ionic’ compounds and compared them with some Ng compounds containing partially covalent bonds (listed in Table 1) [71] with the help of the computational tools discussed in the previous section. While other techniques exist for investigating the nature of bonding, these commonly used and cited tools help eliminate misconceptions among young researchers and advise against the hasty use of terms such as ‘covalent’ and ‘ionic’ to describe a bond. It is important to note that most molecules cannot be exclusively classified as either ‘covalent’ or ‘ionic’.

Table 1 displays the numerical values of different topological descriptors at the concerned BCPs. For the halides of alkali and alkaline earth metals, *ρ*(*r_c_*) and *H*(*r_c_*) values are low and positive, while the Laplacian is positive, and all these values decrease from F to Br. The ratio of *G*(*r_c_*) and *ρ*(*r_c_*) is mostly greater than 1, except for KCl, KBr, CaF_2_, and CaBr_2_, where it is slightly less than 1. These parameters suggest that the interactions are predominantly closed-shell. In contrast, hydrogen halides have higher *ρ*(*r_c_*), negative ▽^2^*ρ*(*r*_c_) and *H*(*r_c_*) values and *G*(*r_c_*)/*ρ*(*r_c_*) ratios that are less than 1 for HCl and greater than 1 for HF and HBr. The initial two parameters suggest a covalent bonding nature, whereas the latter two parameters imply the presence of partially covalent interactions. AIM results for H_2_, N_2_, O_2_, F_2_, Cl_2_, CO, NH_3_, CH_4_, C_2_H_4_, and C_2_H_2_ also imply a partially covalent bond character. For the Ng compounds, the BCPs at the M-N and M-S bonds correspond to slightly larger positive *ρ*(*r_c_*) and ▽^2^*ρ*(*r*_c_) but negative *H*(*r_c_*) values, and a greater *G*(*r_c_*)/*ρ*(*r_c_*) ratio to those of Ng-M bonds. These observations suggest that both bonds possess a partial covalent character, with the Ng-M bond displaying a greater extent of covalency. The ELF values for the halides of alkali and alkaline earth metal are notably low, with Li and Na compounds having the smallest values, followed by the K, Mg, and Ca compounds. Similar results are seen for the Ng compounds as well. These low values indicate minimal covalent character at the BCPs, suggesting predominantly ionic interactions. Conversely, the hydrogen halides, diatomic molecules, hydrocarbons, H_2_O, and NH_3_ have very high ELF values, with most values above 0.8 and some even closer to 1. However, CO is an exception with an ELF value of 0.4. These high ELF values suggest a substantial covalent character at the BCPs.

The values of %*ionic* and %*covalent* characters obtained from using Equations (1) and (2) are provided in Table 2. The percentages of covalent and ionic interactions in the alkali and alkaline earth metal halides range from 78.9% to 93.3% and 6.7% to 21.2%, respectively. They seem to follow Fajans’ rules, where the covalency increases from F to Br for a given metal. In comparison to the metal halides, there is a significant increase in the orbital contribution for the HX molecules, accompanied by a decrease in the electrostatic contribution (∆*E*_elstat_ and ∆*E*_orb_ ranging from 43.4% to 61.5% and 38.5% to 56.5%, respectively). These changes suggest a decrease in ionic character and an increase in covalency, where the covalency even exceeds the ionic character in HCl and HBr. In the common diatomic species such as H_2_, N_2_, O_2_, F_2_, Cl_2_, and CO, the orbital contribution seems to provide the highest stabilization, indicating high covalency (73.5% to 85.1%), with an almost pure covalent bond in the H_2_ molecule. Hence, compounds that are commonly referred to as ‘pure covalent’ still possess a significant electrostatic component. H_2_O, NH_3_, CH_4_, C_2_H_4_, and C_2_H_2_ show high covalency, with an order of N-H < O-H < C-H, as is expected based on the electronegativities of the atoms involved. The π-bonded systems with C-C bonds have a significant ionic component, with acetylene having a greater degree of covalency than ethylene.

The analysis of Ng compounds containing Ng-M bonds (M=Cu, Ag, Au) using EDA involves evaluating two fragmentation schemes, which examine bonds on each side of the metal atoms. In the case of NgMNO_3_ and Ng_2_M_2_SO_4_ species, the Ng-M bonds display comparable contributions from orbital and electrostatic terms, with electrostatic interactions being slightly greater. As a result, these bonds exhibit both a covalent and ionic nature, with % covalency ranging between 38.5% and 46.1%, which is slightly lower. Conversely, the M-N/S bonds are dominated by ∆*E*_elstat_, which accounts for 77.6–86.7% of the interaction and, hence, display a more pronounced % ionic contribution. Even though the dispersion term is the least significant in the total attractive interaction, it is considerably greater in the Ng-M bonds than in the other bonds discussed earlier. As a result, the metal atom is connected on both sides by partially covalent bonds.

## 4. Types of Noble Gas Compounds

Noble gas compounds can be broadly categorized into three groups based on the position of the Ng atom in the compound/complex, viz., non-insertion, insertion, and confined. While the non-insertion compounds contain Ng at one open end of the XY moiety (i.e., NgXY type), the insertion type has the Ng atom inserted within a bond between two elements such that it is flanked by two atoms or groups on either side (i.e., XNgY type of compounds, where the Ng is inserted within the X-Y bond). The stability in the former comes from the polarizing power of the atom adjacent to the Ng (i.e., X) which creates an attractive interaction (donor–acceptor type) between X and Ng. The smaller the size of the X atom, the higher is its polarizing power. There exists an electronegativity difference between the X and Y atoms which produces a dipole and can hence cause the said polarization, the extent of which is higher for ionic systems This XNg moiety is then stabilized by the Y atom, which acts as the counterion. In the case of the insertion complex, however, the stability is kinetics-driven rather than thermodynamics-driven. The reason behind this is the low reactivity of Ng with X and Y compared with the more stable X-Y bond, which has to be destroyed in order to form the Ng complex. Hence, the formation of such XNgY complexes is not thermochemically feasible. However, once it is formed, the high free-energy activation barrier along the reaction path makes the system kinetically stable along that dissociation path.

In the cases of such complexes, it is necessary to consider a significant number of dissociation pathways. Thermochemical analyses reveal that, aside from the two dissociation pathways discussed below, the other dissociation channels are highly or moderately endergonic, indicating that XNgY is stable. The stability of XNgY is determined by two competing dissociation paths: 2B and 3B. While the former involves XNgY splitting into Ng and XY and is typically highly exergonic, the latter results in X, Ng, and Y, and is occasionally exhibits low exergonicity at room temperature. Thus, evaluating the activation energy barrier for these paths is crucial to assessing the stability of XNgY. While single reference-based methods are often reliable for studying the TS of 2B dissociation, computing the barrier of 3B dissociation is more complicated and typically requires multireference treatment. At low temperatures, the most favorable systems are those where the 3B dissociation pathway becomes energetically unfavorable. According to Hu et al. [72], the half-life of Ng compounds with an XNgY structure depends on the energy barrier height. Systems with a minimum energy barrier of 6, 13, and 21 kcal mol^−1^ would correspond to a half-life of approximately 102 s at 100, 200, and 300 K, respectively.

The third category mainly depends on the effects brought about by the confined environment provided by certain cages, e.g., Ng_2_ dimers encapsulated within cages, such as fullerene, B_12_N_12_, B_16_N_16_, C_20_H_20_, B_40_, clathrate hydrates, cucurbit[*n*]uril, octa acids, BN-doped carbon nanotubes, etc., are some of the systems that our group has worked on over the years. Here, the structure, bonding, and reactivity of the encapsulated systems are compared with those before confinement (i.e., in their free states), e.g., as performed for the bonding and movement of Ng_2_ units within fullerene cages [73,74].

## 5. Case Studies of Noble Gas−Noble Metal Binding

Among the bonding of noble gas atoms with other elements of the periodic table, the ones with the noble (or precious) metals, i.e., Cu, Ag, and Au, are of particular interest to us due to the challenges that come with attempting bond formation between two less reactive elements. Research in this field essentially came to the forefront with Pyykkö’s prediction of complexes containing Ng and Au [25,26], followed by pioneering works by several groups in detecting complexes with noble gases and noble metals interacting with each other [29,41,42,43,44,45,75].

**Figure 1:**
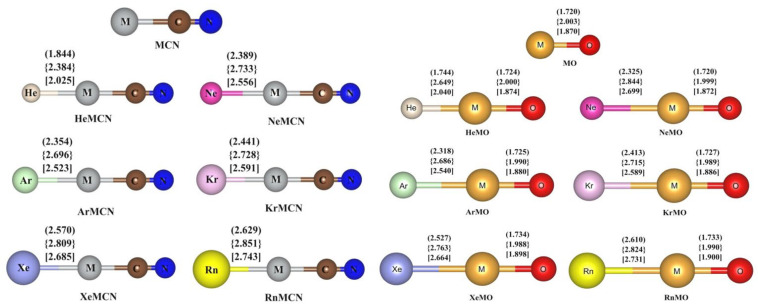
Optimized geometries of the Ng-bound noble metal cyanides (at CCSD(T)/def2-TZVPPD level) and oxides (at CCSD(T)/ccpVTZ/cc-pwCVTZ-PP level) along with the bond distances (in Å) for the Cu, Ag, and Au analogues within parentheses, square bracket, and braces, respectively. [Adapted from Refs. [75,76] with permission from John Wiley and Sons. © 2015 and © 2016 Wiley Periodicals, Inc., respectively].

Over the years, we have performed several theoretical investigations on systems containing possible interactions between noble gases and noble metals. The Ng-binding ability of cyanides, oxides, nitrates, sulphates, and carbonates of the noble metals was investigated by studying the stability and bonding of the non-insertion type complexes NgMY, where M=Cu, Ag, Au, and Y=CN, O, NO_3_, SO_4_, CO_3_ [75,76,77]. The complexes are optimized at different levels, viz., at the CCSD(T)/def2-TZVPPD [78,79,80] and CCSD(T)/ccpVTZ/cc-pwCVTZ-PP [78,81,82,83,84] levels for Y=CN and O, respectively, and at the MPW1B95/def2-TZVP level for Y=NO_3_, SO_4_, CO_3_. The optimized geometries of the cyanides and the oxides are depicted in Figure 1. The dissociation energies (D^0^_BSSE_) of the Ng-M bonds were calculated for NgMCN, NgMO, and Ng-bound metal nitrates, sulphates, and carbonates, considering the zero-point energy (ZPE) and basis set superposition error (BSSE) corrections. The D^0^_BSSE_ values range from 2.7 to 14.6 kcal/mol for NgMCN, with an increase in value from Ar to Rn. Those for the oxides vary from 2.0 to 13.2 kcal/mol, with the same order down the Ng group in the periodic table. For the Ng-bound metal nitrates, sulphates, and carbonates, the values range from 5.1 to 13.2 kcal/mol, 2.3 to 10.1 kcal/mol, and 7.3 to 19.9 kcal/mol, respectively, for the metals moving from Cu to Au. The structural characteristics of both NgMCN and NgMO complexes were compared with those of the experimentally reported NgMX (X=F, Cl, Br) compounds. The results show that AuCN has the highest Ng-binding ability among the noble metal halides, followed by CuCN and AgCN. The ability of CuCN to bind with Ng is greater than that of CuCl and CuBr, but not as high as that of CuF. The orders for the same in the Ag and Au compounds are AgCN ≈ AgF > AgCl > AgBr and AuF > AuCl > AuCN > AuBr, respectively. For the NgMO complexes, the binding power of MO is slightly lower than that of MBr, leading to an order of MF > MCl > MBr > MO. The D_0_ values also increase gradually from Ar to Rn. With respect to thermochemical stability, we see that at room temperature, dissociation of MCN compounds bound to Kr-Rn (except for KrAgCN) and ArAuCN is found to be endergonic (others may need lower temperatures to remain thermochemically stable). That of NgMO complexes into Ng and MO is also endothermic and becomes increasingly so moving from Ar to Rn. The same is calculated for XeAgO, RnAgO, and Kr-Rn-bound CuO and AuO complexes along with most of the sulphates, nitrates, and carbonates, with some exceptions for the Ar and Kr analogues. Lower temperatures may be needed to keep some systems in the bound form.

The bonding picture Is demonstrated in terms of NBO, WBI, EDA, and AIM analyses. The WBI values vary within 0.2–0.3 in the Xe- and Rn-bound MCN complexes. Most of the Ng-M distances, except for all the Ng-Cu bond distances along with that of the Xe and Rn analogues of Ag and Au complexes in NgMCN, are smaller than their respective covalent bond distances. The ∆*E*_HOMO–LUMO_ is higher in these Ng-bound complexes than in their bare counterparts, suggesting higher electronic stability in the former. For the Ng-M bonds in Ar-Rn-bound MO, the WBI ranges are 0.16–0.28, 0.08–0.22, 0.15–0.34 for Cu, Ag, and Au analogues, respectively, with a gradual increase along the group. The same can be said for the Ng-bound complexes of the noble metal sulphates, nitrates, and carbonates. The concerned bond between Ng and M in all the studied complexes can be considered to contain partially covalent and electrostatic types of interactions. The movement of the electron cloud from the noble gas to the middle of the noble gas and noble metal centers suggests an increased likelihood of bond formation. The degree of covalency is higher from Ar to Rn, as evidenced by the increase in the orbital energy term. Both electrostatic and orbital energy values are almost equal in the Ng−Cu bonds, while the orbital contribution is slightly lower than the electrostatic one in NgAgO and NgAuO (excluding ArAuO) (Table S1). In the case of the cyanides, the orbital term is greater in magnitude than the electrostatic term in the NgCuCN complexes, while the reverse is true for the Ag and Au analogues (except for ArAgCN and ArAuCN) (Table S2). All Ng-M bonds in the NgMCN complexes have negative *H*(*r*_c_) values at their bond critical points (except for Ar-Ag), but *G*(*r*_c_)/*ρ*(*r*_c_) < 1 in the Xe/Rn-Cu and Rn-Ag bonds (Table S3). A certain degree of covalency in the concerned bonds of oxides, nitrates, sulphates, and carbonates is further indicated by the electron density analysis (Tables S4 and S5). The optimized geometries of Ng_2_Cu_2_SO_4_ and Ng_2_Ag_2_SO_4_ possess a *D*_2*d*_ symmetry, whereas those of Ng_2_Au_2_SO_4_ and Ng_2_M_2_CO_3_ have *C*_2_ and *C*_2v_ symmetries, respectively. The *D*_0_ values for the Ng-M bond vary in the range 2.5−19.9 kcal/mol in Ng_2_M_2_SO_4_, and 2.3–18.2 kcal/mol in Ng_2_M_2_CO_3_, increasing down the Ng group. The corresponding process for the dissociation of Ng atoms is spontaneous for Ar_2_Cu_2_SO_4_, Ar_2_Ag_2_Y, and Kr_2_Ag_2_Y. Similar to the NgMY systems, the HOMO-LUMO gap also increases in the Ng_2_M_2_Y systems compared with their corresponding bare M_2_Y systems. Except for Ar_2_Ag_2_SO_4_ and Ar_2_Ag_2_SO_4_ all other Ng_2_M_2_Y systems have negative *H*(r_c_) values as calculated from the AIM analysis, suggesting their partial covalent nature (Table S5).

**Figure 2:**
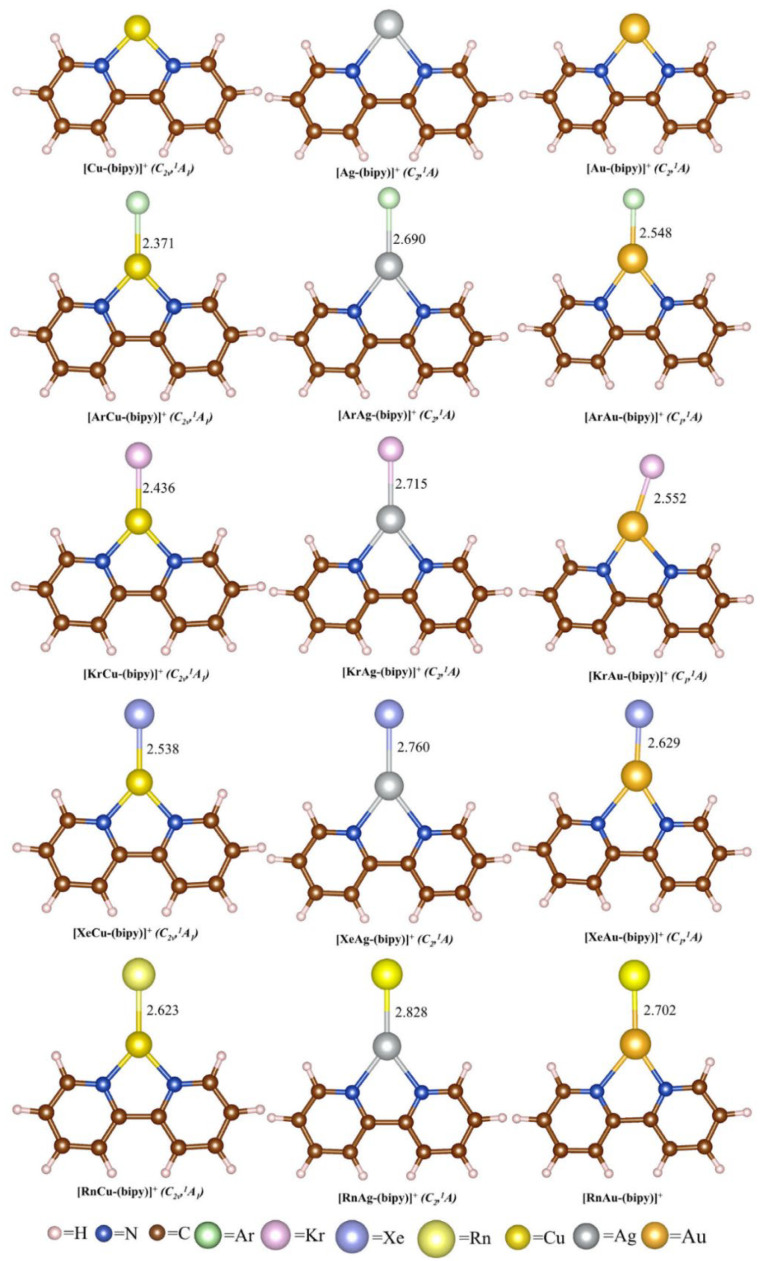
Optimized geometries of the monocationic M-bipyridine complexes at the MPW1B95/cc-pVTZ level of theory, along with the respective bond distances (in Å). [Reprinted from Ref. [85] with permission from John Wiley and Sons. © 2016 Wiley-VCH Verlag GmbH & Co. KGaA, Weinheim].

Apart from the aforementioned complexes, we have also examined the structure, stability, and bonding nature of monocationic M-bipyridine complexes bound to Ng, optimized at the MPW1B95/cc-pVTZ level of theory (Figure 2) [85]. The D^0^_BSSE_ values range from 4.0 to 19.7 kcal/mol, which increase as we move towards heavier Ng atoms. Among the three analogues, the order of Ng binding is Au > Cu > Ag, with the exception of the Ar analogue, where the order is Cu > Au> Ag. We have found that all dissociation processes are increasingly endothermic down the Ng group, and the associated ∆*G* values suggest the thermochemical stability of the Kr-Rn bound complexes. Moving from Ar to Rn, the extent of electron transfer from Ng to the M center increases along with the corresponding WBI values in the Ng-M bonds. Except in the Ar-Ag bond, the *H*(*r_c_*) value is negative (Table S6). Additionally, EDA results show that the attraction between Ng and M centers consists of approximately 45–55% electrostatic and 41–45% orbital interactions, indicating the presence of both covalent and ionic characters (Table S7). An in-depth examination of the orbital term reveals that Ng→Au *σ*-donation and Ng←Au *σ*- and *π*-back donations contribute 61–69% and 27–35% to the total orbital term, respectively.

**Figure 3:**
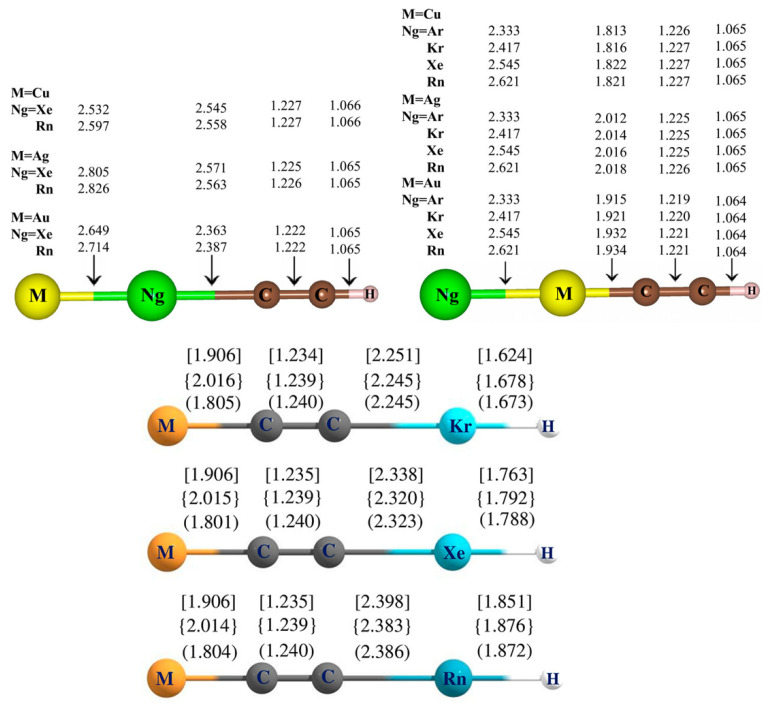
Optimized geometries of the insertion MNgCCH, non-insertion NgMCCH, and MCCNgH complexes, along with the respective bond distances (in Å) for the concerned bonds. The values within the parenthesis, braces, and square brackets are computed for Cu, Ag, and Au, respectively. [Reprinted from Refs. [86,87] with permission from American Chemical Society. © 2017 and © 2018, American Chemical Society, respectively].

Further, the Ng-inserted complexes of MCCH are investigated by considering two possible structures, one where the Ng is inserted within the M-C bond [86] and the other where it is inserted within the C-H bond [87]. The structures are optimized using MPW1B95, [88] MP2 [89], and CCSD(T) [78] methods with basis sets; cc-pVTZ [81,83,90] for H, C, and Ar atoms; and cc-pVTZ-PP with relativistic effective core potential for Kr, Xe, Rn, Cu, Ag, and Au atoms (Figure 3). While for the former, only Xe and Rn were inserted, Kr is also considered for the latter case. The non-insertion isomer, NgMCCH (Ng=Ar-Rn), is found to be viable, especially under low-temperature conditions. The isomerization between the insertion and the non-insertion complex, MNgCCH → NgMCCH, is spontaneous but is kinetically protected by a high free energy activation barrier ranging within 14.0–34.8 kcal mol^−1^, indicating the viability of the insertion complex at room temperature. It is thermochemically stable with respect to other possible dissociation processes, except for a three-body dissociation of the AgXeCCH complex, where a slight lowering of temperature is required to halt the dissociation. Although the Rn analogue is kinetically more stable than the Xe analogue for a given M, the ∆G^╪^ value increases in the order of Ag < Cu < Au for a particular Ng. Additionally, non-inserted NgMCCH compounds that are bound to Cu and Au by Kr-Rn, and to Ag by Xe and Rn, are thermally stable and do not dissociate into Ng and MCCH at 298 K. The M-Ng bonds exhibit high WBI values, i.e., high covalency, whereas the Ng-C bonds are primarily ionic, i.e., best represented as (MNg)^+^(CCH)^−^. EDA results suggest that the interaction between M and Ng is not purely covalent in nature; significant electrostatic interactions contribute to their formation (Table S8). The same is also evident from the electron density descriptors presented in Table S9. Electronic structure principles associated with conceptual DFT, such as maximum hardness [91,92,93] and minimum electrophilicity [93,94,95,96,97] principles, are observed to hold true along the isomerization process MNgCCH → NgMCCH. These compounds represent the first examples of viable species involving an M-Ng-C bonding motif, which expands the current understanding of Ng insertion compounds with unique bonding units. The large ∆G^╪^ values observed in the exergonic dissociation processes of the MNgCCH complex emphasizes the necessity for thorough scrutiny when dealing with species containing the M-Ng-C bonding pattern.

In the cases of the MCCNgH complexes (Figure 3), while the Cu and Ag analogues only prefer to bind with Xe and Rn noble gases, the Au analogue can easily bind with Kr as well. The AuCCH molecule is found to be the most effective in facilitating Ng insertion. While the thermochemically most feasible process (MCCNgH breaking off to produce MCCH and Ng) is significantly hindered by large ∆G^╪^ values at room temperature, the stability of the complex relies upon two other radical dissociation channels (the 2B splitting of MCCNgH to MCC and NgH, and the 3B splitting of MCCNgH to MCC, Ng, and H), which are exergonic at 298 K for Ng=Kr, Xe and M=Cu, Ag, AuCCKrH. The bonding pattern demonstrates that Ng participates in significant covalent interactions with the neighboring atoms (Tables S10 and S11). Additionally, it is feasible to insert two Xe atoms into the Au−C and C−H bonds of AuCCH, producing a kinetically stable AuXeCCXeH molecule. Another isomer of this compound, the XeAuCCXeH, is also stable against splitting into Xe and AuCCXeH. The insertion of three Xe’s, however, is unable to produce a stable complex. These findings thus expand our understanding of the unique bonding present in the Ng insertion compounds.

## 6. Conclusions

Contrary to the popularly used terms, viz., ‘covalent’ and ‘ionic’ bonds, there truly only exist partially covalent bonds comprising both covalent and ionic characters in various compositions, depending upon the electronic structure of the atoms/groups involved. This review article is aimed at studying the nature of the bonds in different compounds, especially those between two less reactive element groups, i.e., noble gases (Ng) and noble metals (M). We have made use of common computational tools such as Natural Bond Orbital (NBO), Quantum Theory of Atoms-in-Molecules (QTAIM), Electron Localization Function (ELF), and Energy Decomposition Analysis (EDA). Investigating possible dissociation channels of the Ng compounds reveals that most of them are endergonic, whereas the exergonic ones have a very high free energy barrier, indicating the stability of the compounds at room temperature. The Ng-binding ability of the compounds containing the noble metals generally follow the order of Au > Cu > Ag. The dissociation channels are increasingly endergonic as the Ng becomes heavier. The WBI values of the bond between Ng and M are mostly lower than 0.5, with a gradual increase along the group. Those in the C-Ng and Ng-H bonds had a range of 0.33–0.44 and 0.60–0.70, respectively. Detailed bonding analysis shows that the contributions from the orbital and electrostatic energy terms are similar in most of the cases (with a range of 45–55%) in the Ng-M bonds of the complexes. This partial covalent nature in these bonds is further indicated by the electron-density analysis. This unusual bonding between noble gases and noble metals is not a simple van der Waals interaction but is instead influenced by a range of factors, including electrostatic, orbital, charge transfer, and polarization effects, which contribute to stabilizing the insertion and non-insertion type of noble gas compounds.

## Figures and Tables

**Table 1:** Electron density descriptors calculated at the BCPs of the concerned bonds [Reprinted from Ref. [71] with permission from Springer Nature. © 2022, Indian Academy of Sciences].

System	BCP	*ρ*(*r*_c_)	∇^2^*ρ*(*r*_c_)	*G*(*r*_c_)	*V*(*r*_c_)	*H*(*r*_c_)	*G*(*r*_c_)/*ρ*(*r*_c_)	ELF
HF	H-F	0.412	−0.339	0.999	−0.105	−0.947	2.425	0.977
HCl	H-Cl	0.310	−0.157	0.143	−0.394	−0.394	0.461	0.999
HBr	H-Br	0.193	−0.420	0.530	−0.211	−0.158	2.747	0.924
LiF	Li-F	0.079	0.705	0.163	−0.149	0.014	2.056	0.062
LiCl	Li-Cl	0.047	0.274	0.065	−0.062	0.003	1.391	0.067
LiBr	Li-Br	0.040	0.198	0.048	−0.046	0.002	1.210	0.070
NaF	Na-F	0.054	0.440	0.101	−0.091	0.009	1.865	0.046
NaCl	Na-Cl	0.035	0.200	0.045	−0.040	0.005	1.279	0.054
NaBr	Na-Br	0.030	0.150	0.034	−0.030	0.004	1.109	0.059
KF	K-F	0.052	0.291	0.071	−0.068	0.002	1.353	0.081
KCl	K-Cl	0.031	0.131	0.031	−0.285	0.002	0.984	0.077
KBr	K-Br	0.027	0.099	0.023	−0.022	0.002	0.868	0.080
MgF_2_	Mg-F	0.082	0.759	0.179	−0.168	0.011	2.177	0.058
MgCl_2_	Mg-Cl	0.055	0.311	0.077	−0.076	0.001	1.392	0.082
MgBr_2_	Mg-Br	0.048	0.222	0.056	−0.057	−0.001	1.171	0.095
CaF_2_	Ca-F	0.045	0.150	0.041	−0.044	−0.003	0.909	0.109
CaCl_2_	Ca-Cl	0.052	0.203	0.054	−0.058	−0.004	1.042	0.129
CaBr_2_	Ca-Br	0.045	0.150	0.041	−0.044	−0.003	0.909	0.138
H_2_	H-H	0.250	−0.982	0.548	−0.246	−0.246	2.195	0.999
N_2_	N-N	0.720	−0.287	0.629	−0.198	−0.135	0.875	0.874
O_2_	O-O	0.546	−0.960	0.492	−0.122	−0.732	0.900	0.820
F_2_	F-F	0.294	0.564	0.287	−0.433	−0.146	0.977	0.628
Cl_2_	Cl-Cl	0.154	−0.205	0.711	−0.147	−0.763	4.634	0.759
CO	C-O	0.508	0.724	0.114	−0.209	−0.955	0.223	0.401
H_2_O	O-H	0.362	−0.241	0.729	−0.749	−0.676	2.015	0.981
NH_3_	N-H	0.337	−0.157	0.603	−0.514	−0.454	1.790	0.984
CH_4_	C-H	0.278	−0.947	0.428	−0.322	−0.279	1.542	0.984
C_2_H_4_	C-C	0.359	−0.112	0.147	−0.574	−0.428	0.409	0.926
C_2_H_2_	C-C	0.426	−0.125	0.314	−0.940	−0.626	0.737	0.830
ArCuNO_3_	Ar-Cu	0.042	0.209	0.056	−0.059	−0.003	1.312	0.066
	Cu-N	0.069	0.322	0.096	−0.111	−0.015	1.397	0.106
KrCuNO_3_	Kr-Cu	0.053	0.188	0.053	−0.058	−0.006	1.000	0.096
	Cu-N	0.068	0.320	0.095	−0.110	−0.015	1.394	0.106
XeCuNO_3_	Xe-Cu	0.049	0.157	0.047	−0.055	−0.008	0.957	0.139
	Cu-N	0.068	0.317	0.094	−0.109	−0.148	1.391	0.105
RnCuNO_3_	Rn-Cu	0.047	0.136	0.042	−0.049	−0.007	0.889	0.149
	Cu-O	0.067	0.315	0.093	−0.108	−0.015	1.389	0.104
ArAgNO_3_	Ar-Ag	0.026	0.110	0.028	−0.028	0.000	1.053	0.055
	Ag-N	0.057	0.247	0.068	−0.075	−0.007	1.202	0.111
KrAgNO_3_	Kr-Ag	0.033	0.116	0.031	−0.339	−0.002	0.943	0.090
	Ag-N	0.057	0.245	0.068	−0.074	−0.007	1.202	0.110
XeAgNO_3_	Xe-Ag	0.039	0.111	0.033	−0.037	−0.005	0.833	0.137
	Ag-N	0.056	0.244	0.068	−0.074	−0.007	1.201	0.110
RnAgNO_3_	Rn-Ag	0.039	0.100	0.030	−0.036	−0.005	0.776	0.153
	Ag-N	0.056	0.243	0.067	−0.074	−0.006	1.200	0.109
Ar_2_Ag_2_SO_4_	Ar-Ag	0.024	0.102	0.025	−0.025	0.002	1.037	0.051
	Ag-S	0.055	0.245	0.067	−0.073	−0.006	1.221	0.104
Kr_2_Ag_2_SO_4_	Kr-Ag	0.032	0.110	0.030	−0.318	−0.214	0.937	0.085
	Ag-S	0.055	0.245	0.067	−0.073	−0.006	1.220	0.104
Xe_2_Ag_2_SO_4_	Xe-Ag	0.038	0.109	0.032	−0.036	−0.004	0.834	0.132
	Ag-S	0.055	0.243	0.067	−0.073	−0.006	1.218	0.104
Rn_2_Ag_2_SO_4_	Rn-Ag	0.038	0.098	0.030	−0.035	−0.005	0.778	0.148
	Ag-S	0.055	0.242	0.066	−0.072	−0.006	1.217	0.103

**Table 2:** The interaction energy components (in kcal mol^−1^) calculated at the B3LYP-D3(BJ)/TZ2P level obtained from EDA along with the %covalent and ionic characters of the concerned bonds [Reprinted from Ref. [71] with permission from Springer Nature. © 2022, Indian Academy of Sciences].

System	Bonds	Δ*E*_pauli_	Δ*E*_elstat_	Δ*E*_orb_	Δ*E*_disp_	Δ*E*_int_	%*Covalent*	%*Ionic*
HF	H-F	0.0	−232.76	−145.92	−0.11	−378.79	38.53	61.47
HCl	H-Cl	0.0	−159.91	−178.63	−0.35	−338.89	52.76	47.24
HBr	H-Br	−0.01	−141.69	−184.5	−0.43	−326.61	56.56	43.44
LiF	Li-F	41.38	−210.53	−24.01	−0.28	−193.44	10.24	89.76
LiCl	Li-Cl	29.16	−160.53	−26.33	−0.91	−158.62	14.09	85.91
LiBr	Li-Br	27.72	−147.42	−26.89	−1.07	−147.66	15.43	84.57
NaF	Na-F	31.09	−179.91	−12.91	−0.34	−162.06	6.70	93.30
NaCl	Na-Cl	25.45	−146.21	−14.95	−1.03	−136.74	9.28	90.72
NaBr	Na-Br	24.6	−137.15	−16.36	−1.19	−130.09	10.66	89.34
KF	K-F	39.61	−163.92	−21.37	−0.43	−146.11	11.53	88.47
KCl	K-Cl	29.18	−132.33	−15.93	−1.21	−120.29	10.74	89.26
KBr	K-Br	28.28	−125.09	−15.59	−1.36	−113.75	11.08	88.92
MgF_2_	Mg-F	95.84	−734.88	−82.7	−0.81	−722.56	10.12	89.88
MgCl_2_	Mg-Cl	77.01	−582.58	−120.12	−2.52	−628.21	17.09	82.91
MgBr_2_	Mg-Br	69.11	−528.17	−141.21	−2.93	−603.2	21.10	78.90
CaF_2_	Ca-F	121.9	−666.31	−79.32	−0.98	−624.71	10.64	89.36
CaCl_2_	Ca-Cl	101.73	−542.27	−92.56	−2.82	−535.92	14.58	85.42
CaBr_2_	Ca-Br	95.85	−501.53	−103.75	−0.08	−509.51	17.14	82.86
H_2_	H-H	226.24	6.19	−410.2	−0.09	−177.86	101.53	−1.53
N_2_	N-N	1683.65	−320.32	−1830.98	−0.47	−468.11	85.11	14.89
O_2_	O-O	956.56	−239.07	−849.78	−0.29	−132.58	78.04	21.96
F_2_	F-F	335.12	−98.82	−381.46	−0.17	−145.34	79.42	20.58
Cl_2_	Cl-Cl	298.94	−109.56	−303.5	−1.29	−115.41	73.48	26.52
CO	C-O	1362.11	−277.75	−1502.04	−0.46	−418.14	84.39	15.61
H_2_O	O-H	409.15	−69.44	−534.56	−0.21	−195.04	88.50	11.50
NH_3_	N-H	418.92	−84.04	−514.21	−0.3	−179.63	85.95	14.05
CH_4_	C-H	387.57	−55.77	−511.01	−0.33	−179.53	90.16	9.84
C_2_H_4_	C-C	1154.17	−453.35	−908.61	−1.42	−209.21	66.71	33.29
C_2_H_2_	C-C	1373.8	−463.1	−1210.62	−0.89	−300.81	72.33	27.67
ArCuNO_3_	Ar-Cu	18	−13.27	−11.36	−1.38	−8.01	46.12	53.88
	Cu-N	67.77	−186.74	−47.74	−2.15	−168.87	20.36	79.64
KrCuNO_3_	Kr-Cu	22.86	−17.78	−15.01	−1.7	−11.63	45.78	54.22
	Cu-N	67.03	−182.95	−47.83	−2.24	−166	20.73	79.27
XeCuNO_3_	Xe-Cu	28.91	−23.59	−18.59	−2.19	−15.45	44.07	55.93
	Cu-N	66.82	−178.15	−48.15	−2.36	−161.84	21.28	78.72
RnCuNO_3_	Rn-Cu	29.16	−24.27	−18.69	−2.31	−16.12	43.51	56.49
	Cu-O	66.98	−175.24	−48.22	−2.41	−158.89	21.58	78.42
ArAgNO_3_	Ar-Ag	81.38	−6.34	−5.43	−1.46	68.15	46.13	53.87
	Ag-N	60.16	−167.49	−38.87	−2.38	−148.59	18.84	81.16
KrAgNO_3_	Kr-Ag	14.98	−11.05	−9.14	−1.8	−7.01	45.27	54.73
	Ag-N	59.13	−164.45	−38.93	−2.5	−146.74	19.14	80.86
XeAgNO_3_	Xe-Ag	23.11	−17.96	−13.1	−2.32	−10.26	42.18	57.82
	Ag-N	58.81	−160.71	−39.21	−2.57	−143.68	19.61	80.39
RnAgNO_3_	Rn-Ag	25.04	−19.98	−13.96	−2.43	−11.33	41.13	58.87
	Ag-N	58.55	−158.24	−39.25	−2.61	−141.55	19.87	80.13
Ar_2_Ag_2_SO_4_	Ar-Ag	8.27	−5.82	−4.55	−1.5	−3.6	43.88	56.12
	Ag-S	135.09	−522.47	−73.49	−6.9	−467.78	12.33	87.67
Kr_2_Ag_2_SO_4_	Kr-Ag	14.25	−10.55	−8.04	−1.88	−6.22	43.25	56.75
	Ag-S	134.31	−511.84	−76.42	−7.27	−461.22	12.99	87.01
Xe_2_Ag_2_SO_4_	Xe-Ag	22.95	−17.88	−11.83	−2.45	−9.2	39.82	60.18
	Ag-S	133.17	−496.82	−80.74	−7.52	−451.9	13.98	86.02
Rn_2_Ag_2_SO_4_	Rn-Ag	24.84	−19.85	−12.44	−2.59	−10.05	38.53	61.47
	Ag-S	132.96	−488.06	−82.97	−7.65	−445.72	14.53	85.47

## Data Availability

Data is available from the authors on reasonable request.

## Supplementary Materials

The following supporting information can be downloaded at: https://www.mdpi.com/article/10.3390/molecules28073253/s1, Table S1: EDA results of NgMO (M = Cu, Ag, Au) complexes considering Ng as one fragment and MO as another fragment at the PBE-D3/QZ4P//CCSD(T)/VTZ level. All energy terms are in kcal/mol. (The values within the parentheses are in percentage and show the contribution toward the total attractive interaction, ∆Eelstat + ∆Eorb + ∆Edisp). [Reprinted from Ref. [76] with permission from John Wiley and Sons. © 2015 Wiley Periodicals, Inc.]; Table S2: EDA results of the NgMCN (M = Cu, Ag, Au) clusters with Ng and MCN as fragments calculated at the PBE-D3/QZ4P//CCSD(T)/def2-TZVPPD level. All energy terms are in kcal/mol. (The percentage values within the parentheses show the contribution toward the total attractive interaction, ∆Eelstat + ∆Eorb + ∆Edisp). [Reprinted from Ref. [75] with permission from John Wiley and Sons. © 2015 Wiley Periodicals, Inc.]; Table S3: Electron density descriptors (au) at the bond critical points (BCPs) in between Ng and M atoms in NgMCN obtained from the wave functions generated at the MP2/def2-TZVPPD/WTBS//CCSD(T)/def2-TZVPPD level (All electron WTBS basis set is used only for Ag, Au, Xe, and Rn). [Reprinted from Ref. [75] with permission from John Wiley and Sons. © 2015 Wiley Periodicals, Inc.]; Table S4: Electron density descriptors (au) at the BCPs in between Ng and M centers obtained from the wave functions generated at the MP2/cc-pVTZ/WTBS//CCSD(T)/VTZ level (All electron WTBS basis set is used only for Ag, Au, Xe, and Rn). [Reprinted from Ref. [76] with permission from John Wiley and Sons. © 2015 Wiley Periodicals, Inc.]; Table S5: Different topological descriptors (au) at the bond critical points (BCP) in between Ng and M atoms in NgMY and Ng2M2Y (M = Cu, Ag; Y = NO3, SO4, CO3) complexes at the MPW1B95/def2-TZVP level. [Reprinted from Ref. [77] with permission from Springer Nature. © 2016, Indian Academy of Sciences]; Table S6: Topological descriptors (au) at the line critical point between Ng and M atoms in [NgM-(bipy)]+ obtained from wave function generated at the MPW1B95/cc-pVTZ/WTBS//MPW1B95/cc-pVTZ level (All electron WTBS basis set is used only for Cu, Ag, Au, Kr, Xe and Rn). [Reprinted from Ref. [85] with permission from John Wiley and Sons. © 2016 Wiley-VCH Verlag GmbH & Co. KGaA, Weinheim]; Table S7: Energy decomposition analysis (EDA) results of the [NgM-(bipy)]+ complexes taking Ng as one fragment and [M-(bipy)]+ as another, studied at the BLYP-D3(BJ)/QZ4P//MPW1B95/cc-pVTZ level. All the energy terms are in kcal/mol. The percentage values within the parentheses show the contribution towards the total attractive interaction, ∆Eelstat + ∆Eorb + ∆Edisp. [Reprinted from Ref. [85] with permission from John Wiley and Sons. © 2016 Wiley-VCH Verlag GmbH & Co. KGaA, Weinheim]; Table S8: EDA results of MNgCCH and NgMCCH at the PBE-D3(BJ)/QZ4P//CCSD(T)/cc-pVTZ level. All energy values are in kcal/mol. The values in parentheses are percentage contribution toward the total attraction, ∆Eelstat + ∆Eorb + ∆Edisp. [Reprinted from Ref. [86] with permission from American Chemical Society. © 2017, American Chemical Society]; Table S9: Electron density descriptors (au) at the BCPs of M-Ng and Ng-C bonds in MNgCCH compounds obtained from the wave functions generated at the MP2/ccpVTZ/WTBS//CCSD(T)/cc-pVTZ level (WTBS for Cu, Ag, Au, Xe and Rn atoms). [Reprinted from Ref. [86] with permission from American Chemical Society. © 2017, American Chemical Society]; Table S10: Electron density descriptors (in au) calculated at the MP2/cc-pVTZ/WTBS//CCSD(T)/VTZ level. [Reprinted from Ref. [87] with permission from American Chemical Society. © 2018, American Chemical Society]; Table S11: EDA Results of MCCNgH at the PBE-D3/QZ4P//CCSD(T)/VTZ Level. All energy values are in kcal/mol. The values in parentheses are the percentage contribution toward the total attraction, ∆Eelstat + ∆Eorb + ∆Edisp. [Reprinted from Ref. [87] with permission from American Chemical Society. © 2018, American Chemical Society]

## References

[1] Kossel W. (1916). Über Molekülbildung als Frage des Atombaus. Ann. Der Phys..

[2] Von Antropoff A. (1924). Die Wertigkeit der Edelgase und ihre Stellung im periodischen System. II. Angew. Chem. Int. Ed..

[3] Pauling L. (1933). The formulas of antimonic acid and the antimonates. J. Am. Chem. Soc..

[4] Bartlett N., Lohmann D. (1962). 1005. Fluorides of the noble metals. Part II. Dioxygenyl hexafluoroplatinate (V), O2+[PtF6]-. J. Am. Chem. Soc..

[5] Bartlett N. (1962). Xenon hexafluoroplatinate (V) Xe^+^[PtF_6_]^-^. Proc. Chem. Soc. Lond..

[6] Hargittai I. (2009). Neil Bartlett and the first noble-gas compound. Struct. Chem..

[7] Grandinetti F. (2022). 60 years of chemistry of the noble gases. Nature.

[8] Graham L., Graudejus O., Jha N.K., Bartlett N. (2000). Concerning the nature of XePtF6. Coord. Chem. Rev..

[9] Streng A., Kirshenbaum A., Streng L., Grosse A., Hyman H.H. (1963). Preparation of Rare-Gas Fluorides and Oxyfluorides by the Electric Discharge Method and their Properties. Noble Gas Compounds.

[10] Lehmann J.F., Mercier H.P., Schrobilgen G.J. (2002). The chemistry of krypton. Coord. Chem. Rev..

[11] Claassen H.H., Selig H., Malm J.G. (1962). Xenon tetrafluoride. J. Am. Chem. Soc..

[12] Slivnik J., Brcic B., Volavsek B., Marsel J., Vrscaj V., Smalc A., Frlec B., Zemljic Z. (1962). Über die Synthese von XeF6. Croat. Chem. Acta.

[13] Turner J., Pimentel G.C. (1963). Krypton fluoride: Preparation by the matrix isolation technique. Science.

[14] Nelson L.Y., Pimentel G.C. (1967). Infrared detection of xenon dichloride. Inorg. Chem..

[15] Bartlett N., Wechsberg M. (1951). The Xenon Difluoride Complexes XeF_2_ · XeOF_4_; XeF_2_ · XeF_6_ · AsF_5_ and XeF_2_ · 2XeF_6_ · 2AsF_5_ and Their Relevance to Bond Polarity and Fluoride Ion Donor Ability of XeF_2_ and XeF_6_. Z. Anorg. Allg. Chem..

[16] Holloway J.H., Hope E.G. (1998). Recent advances in noble-gas chemistry. Adv. Inorg. Chem..

[17] Stein L. (1970). Ionic radon solutions. Science.

[18] Khriachtchev L., Pettersson M., Runeberg N., Lundell J., Räsänen M. (2000). A stable argon compound. Nature.

[19] Frenking G. (2000). Another noble gas conquered. Nature.

[20] Wang Q., Wang X. (2013). Infrared Spectra of NgBeS (Ng = Ne, Ar, Kr, Xe) and BeS2 in Noble-Gas Matrices. J. Phys. Chem. A.

[21] Zhang Q., Chen M., Zhou M., Andrada D.M., Frenking G. (2014). Experimental and Theoretical Studies of the Infrared Spectra and Bonding Properties of NgBeCO_3_ and a Comparison with NgBeO (Ng = He, Ne, Ar, Kr, Xe). J. Phys. Chem. A.

[22] Yu W., Liu X., Xu B., Xing X., Wang X. (2016). Infrared Spectra of Novel NgBeSO2 Complexes (Ng = Ne, Ar, Kr, Xe) in Low Temperature Matrixes. J. Phys. Chem. A.

[23] Zhang Q., Li W.L., Zhao L., Chen M., Zhou M., Li J., Frenking G. (2017). A Very Short Be-Be Distance but No Bond: Synthesis and Bonding Analysis of Ng-Be2O2-Ng0 (Ng, Ng = Ne, Ar, Kr, Xe). Chem. Eur. J..

[24] Dong X., Oganov A.R., Goncharov A.F., Stavrou E., Lobanov S., Saleh G., Qian G.-R., Zhu Q., Gatti C., Deringer V.L. (2017). A stable compound of helium and sodium at high pressure. Nat. Chem..

[25] Pyykko P. (1995). Predicted chemical bonds between rare gases and Au. J. Am. Chem. Soc..

[26] Schr€oder D., Schwarz H., Hrusak J., Pyykko P. (1998). Cationic gold (I) complexes of xenon and of ligands containing the donor atoms oxygen, nitrogen, phosphorus, and sulfur. Inorg. Chem..

[27] Read J.P., Buckingham A.D. (1997). Covalency in ArAu^+^ and Related Species?. J. Am. Chem. Soc..

[28] Evans C.J., Lesarri A., Gerry M.C.L. (2000). Noble Gas—Metal Chemical Bonds. Microwave Spectra, Geometries, and Nuclear Quadrupole Coupling Constants of Ar-AuCl and Kr-AuCl. J. Am. Chem. Soc..

[29] Seidel S., Seppelt K. (2000). Xenon as a complex ligand: The tetra Xenono Gold (II) cation in AuXe_4_^2+^(Sb_2_F_11_^−^)_2_. Science.

[30] Cooke S.A., Gerry M.C.L. (2004). Insights into the Xenon–Silver Halide Interaction from a Rotational Spectroscopic Study of XeAgF and XeAgCl. Phys. Chem. Chem. Phys..

[31] Cooke S.A., Gerry M.C.L. (2004). XeAuF. J. Am. Chem. Soc..

[32] Thomas J.M., Walker N.R., Cooke S.A., Gerry M.C.L. (2004). Microwave Spectra and Structures of KrAuF, KrAgF, and KrAgBr; 83Kr Nuclear Quadrupole Coupling and the Nature of Noble Gas−Noble Metal Halide Bonding. J. Am. Chem. Soc..

[33] Michaud J.M., Cooke S.A., Gerry M.C.L. (2004). Rotational Spectra, Structures, Hyperfine Constants, and the Nature of the Bonding of KrCuF and KrCuCl. Inorg. Chem..

[34] Michaud J.M., Gerry M.C.L. (2006). XeCu Covalent Bonding in XeCuF and XeCuCl, Characterized by Fourier Transform Microwave Spectroscopy Supported by Quantum Chemical Calculations. J. Am. Chem. Soc..

[35] Lantto P., Vaara J. (2006). Calculations of nuclear quadrupole coupling in noble gas–noble metal fluorides: Interplay of relativistic and electron correlation effects. J. Chem. Phys..

[36] Mou C.H., Witek H.A. (2008). Theoretical study of noble-gas containing metal halides. J. Chem. Phys..

[37] Zou W., Liu Y., Boggs J.E. (2009). Theoretical study of RgMF (Rg= He, Ne; M= Cu, Ag, Au): Bonded structures of helium. Chem. Phys. Lett..

[38] Chen R., Zhu H., Xie D.Q., GuoSen G.S. (2009). Theoretical prediction of the noble gas complexes HeAuF and NeAuF. Sci. Chin. Ser. B Chem..

[39] Evans C.J., Wright T.G., Gardner A.M. (2010). Geometries and Bond Energies of the He− MX, Ne− MX, and Ar− MX (M= Cu, Ag, Au; X= F, Cl) Complexes. J. Phys. Chem. A.

[40] Beyhan S.M., Gotz A.W., Jacob C.R., Visscher L. (2010). The weak covalent bond in NgAuF (Ng = Ar, Kr, Xe): A challenge for subsystem density functional theory. J. Chem. Phys..

[41] Wang X., Andrews L., Brosi F., Riedel S. (2013). Matrix Infrared Spectroscopy and Quantum-Chemical Calculations for the Coinage-Metal Fluorides: Comparisons of Ar-AuF, Ne-AuF, and Molecules MF_2_ and MF_3_. Chem. Eur. J..

[42] Zhang P.X., Zhao Y.F., Hao F.Y., Zhang G.H., Song X.D., Li X.Y. (2008). Bonding analysis for NgMOH (Ng= Ar, Kr and Xe; M= Cu and Ag). Mol. Phys..

[43] Zhang P.X., Zhao Y.F., Hao F.Y., Li X.Y. (2008). Bonding analysis for NgAuOH (Ng= Kr, Xe). Int. J. Quantum Chem..

[44] Ghanty T.K. (2005). Insertion of noble-gas atom (Kr and Xe) into noble-metal molecules (AuF and AuOH): Are they stable?. J. Chem. Phys..

[45] Ghanty T.K. (2006). How strong is the interaction between a noble gas atom and a noble metal atom in the insertion compounds MNgF (M=Cu and Ag, and Ng=Ar, Kr, and Xe)?. J. Chem. Phys..

[46] Glendening E.D., Landis C.R., Weinhold F. (2013). NBO6.0: Natural bond orbital analysis program. J. Comput. Chem..

[47] Wiberg K.B. (1968). Application of the Pople-Santry-Segal CNDO Method to the Cyclopropylcarbinyl and Cyclobutyl Cation and to Bicyclobutane. Tetrahedron.

[48] Bader R.F. (1985). Atoms in molecules. Acc. Chem. Res..

[49] Bader R.F.W. (1990). Atoms in Molecules: A Quantum Theory.

[50] Bader R.F.W. (1975). Molecular fragments or chemical bonds. Acc. Chem. Res..

[51] Silvi B., Savin A. (1994). Classification of chemical bonds based on topological analysis of electron localization functions. Nature.

[52] Becke A.D., Edgecombe K.E. (1990). A simple measure of electron localization in atomic and molecular systems. J. Chem. Phys..

[53] Fonseca G.C., Handgraaf J.W., Baerends E.J., Bickelhaupt F.M. (2004). Voronoi deformation density (VDD) charges: Assessment of the Mulliken, Bader, Hirshfeld, Weinhold, and VDD methods for charge analysis. J. Comput. Chem..

[54] Haaland A., Helgaker T.U., Ruud K., Shorokhov D.J. (2000). Should Gaseous BF_3_ and SiF_4_Be Described as Ionic Compounds?. J. Chem. Educ..

[55] De Proft F., Van Alsenoy C., Peeters A., Langenaeker W., Geerlings P. (2002). Atomic charges, dipole moments, and Fukui functions using the Hirshfeld partitioning of the electron density. J. Comput. Chem..

[56] Jensen F. (1999). Introduction To Computational Chemistry.

[57] Brom J.M., Schmitz B.J., Thompson J.D., Cramer C.J., Truhlar D.G. (2003). A Class IV Charge Model for Boron Based on Hybrid Density Functional Theory. J. Phys. Chem. A.

[58] Bader R.F.W., Matta C.F. (2013). Atoms in molecules as non-overlapping, bounded, space-filling open quantum systems. Found. Chem..

[59] Bader R.F.W., Zou P.F. (1992). An atomic population as the expectation value of a quantum observable. Chem. Phys. Lett..

[60] Borocci S., Grandinetti F., Sanna N. (2022). Noble-gas compounds: A general procedure of bonding analysis. J. Chem. Phys..

[61] Makarewicz E., Gordon A.J., Berski S. (2015). Nature of the Bonding in the AuNgX (Ng = Ar, Kr, Xe; X = F, Cl, Br, I) Molecules. Topological Study on Electron Density and the Electron Localization Function (ELF). J. Phys. Chem. A.

[62] Borocci S., Giordani M., Grandinetti F. (2015). Bonding Motifs of Noble-Gas Compounds as Described by the Local Electron Energy Density. J. Phys. Chem. A.

[63] Borocci S., Grandinetti F., Nunzi F., Sanna N. (2020). Classifying the chemical bonds involving the noble-gas atoms. New J. Chem..

[64] Michalak A., Mitoraj M., Ziegler T. (2008). Bond orbitals from chemical valence theory. J. Phys. Chem. A.

[65] Mitoraj M.P., Michalak A., Ziegler T. (2009). A combined charge and energy decomposition scheme for bond analysis. J. Chem. Theory Comput..

[66] Pan S., Saha R., Chattaraj P.K. (2015). Exploring the nature of silicon-noble gas bonds in H_3_SiNgNSi and HSiNgNSi compounds (Ng = Xe, Rn). Int. J. Mol. Sci..

[67] Tonner R., Frenking G. (2008). Divalent carbon (0) chemistry, part 1: Parent compounds. Chem. Eur. J..

[68] Frisch M.J., Trucks G.W., Schlegel H.B., Scuseria G.E., Robb M.A., Cheeseman J.R., Scalmani G., Barone V., Mennucci B., Petersson G.A. (2010). Gaussian 09, Revision C.01.

[69] Lu T., Chen F. (2012). Multiwfn: A multifunctional wavefunction analyzer. J. Comput. Chem..

[70] Baerends E.J., Ziegler T., Autschbach J., Bashford D., B_erces A., Bickelhaupt F.M., Bo C., Boerrigter P.M., Cavallo L., Chong D.P. (2013). ADF2013.01; SCM, Theoretical Chemistry.

[71] Pal R., Patra S.G., Chattaraj P.K. (2022). Can a chemical bond be exclusively covalent or ionic?. J. Chem. Sci..

[72] Li T.H., Liu Y.L., Lin R.J., Yeh T.Y., Hu W.P. (2007). On the stability of noble gas molecules. Chem. Phys. Lett..

[73] Krapp A., Frenking G. (2007). Is this a chemical bond? a theoretical study of Ng_2_@C_60_ (Ng= He, Ne, Ar, Kr, Xe). Chem. Eur. J..

[74] Khatua M., Pan S., Chattaraj P.K. (2014). Movement of Ng_2_ molecules confined in a C_60_ cage: An ab initio molecular dynamics study. Chem. Phys. Lett..

[75] Pan S., Gupta A., Saha R., Merino G., Chattaraj P.K. (2015). A coupled-cluster study on the noble gas binding ability of metal cyanides versus metal halides (metal = Cu, Ag, Au). J. Comput. Chem..

[76] Pan S., Saha R., Kumar A., Gupta A., Merino G., Chattaraj P.K. (2016). A noble interaction: An assessment of noble gas binding ability of metal oxides (metal = Cu, Ag, Au). Int. J. Quantum Chem..

[77] Ghara M., Pan S., Deb J., Kumar A., Sarkar U., Chattaraj P.K. (2016). A computational study on structure, stability and bonding in Noble Gas bound metal Nitrates, Sulfates and Carbonates (metal = Cu, Ag, Au). J. Chem. Sci..

[78] Pople J.A., Head-Gordon M., Raghavachari K. (1987). Quadratic configuration interaction. A general technique for determining electron correlation energies. J. Chem. Phys..

[79] Weigend F., Ahlrichs R. (2005). Balanced basis sets of split valence, triple zeta valence and quadruple zeta valence quality for H to Rn: Design and assessment of accuracy. Phys. Chem. Chem. Phys..

[80] Weigend F. (2006). Accurate Coulomb-fitting basis sets for H to Rn. Phys. Chem. Chem. Phys..

[81] Dunning Jr T.H. (1989). Gaussian basis sets for use in correlated molecular calculations. I. The atoms boron through neon and hydrogen. J. Chem. Phys..

[82] Kendall R.A., Dunning T.H., Harrison R.J. (1992). Electron affinities of the first-row atoms revisited. Systematic basis sets and wave functions. J. Chem. Phys..

[83] Woon D.E., Dunning T.H. (1993). Gaussian basis sets for use in correlated molecular calculations. III. The atoms aluminum through argon. J. Chem. Phys..

[84] Peterson K.A., Woon  D.E., Dunning T.H. (1994). Benchmark calculations with correlated molecular wave functions. IV. The classical barrier height of the H+ H2→ H2+ H reaction. J. Chem. Phys..

[85] Jana G., Saha R., Pan S., Kumar A., Merino G., Chattaraj P.K. (2016). Noble gas binding ability of metal-bipyridine monocationic complexes (metal = Cu, Ag, Au): A computational study. ChemistrySelect.

[86] Jana G., Pan S., Merino G., Chattaraj P.K. (2017). MNgCCH (M = Cu, Ag, Au; Ng = Xe, Rn): The First Set of Compounds with M–Ng–C Bonding Motif. J. Phys. Chem. A.

[87] Jana G., Pan S., Merino G., Chattaraj P.K. (2018). Noble Gas Inserted Metal Acetylides (Metal = Cu, Ag, Au). J. Phys. Chem. A.

[88] Zhao Y., Truhlar D.G. (2004). Hybrid Meta Density Functional Theory Methods for Thermochemistry, Thermochemical Kinetics, and Noncovalent Interactions: The MPW1B95 and MPWB1K Models and Comparative Assessments for Hydrogen Bonding and van der Waals Interactions. J. Phys. Chem. A.

[89] Moller C., Plesset M.S. (1934). Note on an Approximation Treatment for Many-Electron Systems. Phys. Rev..

[90] Woon D.E., Dunning Jr T.H. (1995). Gaussian basis sets for use in correlated molecular calculations. V. Core-valence basis sets for boron through neon. J. Chem. Phys..

[91] Pearson R.G. (1993). The principle of maximum hardness. Acc. Chem. Res..

[92] Pearson R.G. (1999). Maximum Chemical and Physical Hardness. J. Chem. Educ..

[93] Pan S., Solà M., Chattaraj P.K. (2013). On the validity of the maximum hardness principle and the minimum electrophilicity principle during chemical reactions. J. Phys. Chem. A.

[94] Chamorro E., Chattaraj P.K., Fuentealba P. (2003). Variation of the electrophilicity index along the reaction path. J. Phys. Chem. A.

[95] Parthasarathi R., Elango M., Subramanian V., Chattaraj P.K. (2005). Variation of electrophilicity during molecular vibrations and internal rotations. Theor. Chem. Acc..

[96] Chattaraj P.K., Giri S. (2007). A minimum electrophilicity perspective of the HSAB principle. Indian J. Phys..

[97] Miranda-Quintana R.A., Chattaraj P.K., Ayers P.W. (2017). Finite temperature grand canonical ensemble study of the minimum electrophilicity principle. J. Chem. Phys..

