# Supplementary material for: On the Nature of the Partial Covalent Bond between Noble Gas Elements and Noble Metal Atoms
Source: Molecules. 2023 Apr 5;28(7):3253. doi: 10.3390/molecules28073253 (PMC10096529; doi:10.3390/molecules28073253)
Supplement: Supplementary file 1 [file molecules-28-03253-s001.zip › molecules-2303928-supplementary.pdf]

# On the Nature of the Partial Covalent Bond between Noble Gas Elements and Noble Metal Atoms

**Ranita Pal [1] and Pratim Kumar Chattaraj [2,*]**

[1] Advanced Technology Development Centre, Indian Institute of Technology, Kharagpur 721302, India
[2] Department of Chemistry, Indian Institute of Technology, Kharagpur 721302, India
[*] Correspondence: pkc@chem.iitkgp.ac.in

## SUPPLIMENTARY INFORMATION

**Table S1**. EDA results of NgMO (M = Cu, Ag, Au) complexes considering Ng as one fragment and MO as another fragment at the PBE-D3/QZ4P//CCSD(T)/VTZ level. All energy terms are in kcal/mol. (The values within the parentheses are in percentage and show the contribution toward the total attractive interaction, $\Delta E_{elstat}$ + $\Delta E_{orb}$ + $\Delta E_{disp}$). [Reprinted from Ref. [76] with permission from John Wiley and Sons. © 2015 Wiley Periodicals, Inc.]

| Systems | $\Delta E_{int}$ | $\Delta E_{elstat}$ | $\Delta E_{orb}$ | $\Delta E_{Pauli}$ | $\Delta E_{disp}$ |
|---|---|---|---|---|---|
| ArCuO | −6.66 | −15.4 (49.3) | −15.7 (50.2) | 24.6 | −0.1 (0.4) |
| KrCuO | −8.91 | −19.3 (50.3) | −18.9 (49.2) | 29.5 | −0.2 (0.4) |
| XeCuO | −12.93 | −25.2 (49.9) | −25.1 (49.7) | 37.5 | −0.2 (0.4) |
| RnCuO | −14.07 | −25.3 (49.6) | −25.4 (50.0) | 36.9 | −0.22 (0.4) |
| ArAgO | −4.44 | −8.0 (50.6) | −7.6 (47.7) | 11.4 | −0.2 (1.7) |
| KrAgO | −6.63 | −12.8 (52.9) | −11.1 (45.9) | 17.5 | −0.29 (1.2) |
| XeAgO | −10.14 | −21.3 (55.5) | −16.9 (43.8) | 28.3 | −0.29 (0.8) |
| RnAgO | −11.56 | −23.1 (55.8) | −17.9 (43.3) | 29.9 | −0.37 (0.9) |
| ArAuO | −6.66 | −17.0 (49.3) | −17.2 (40.1) | 27.7 | −0.21 (0.6) |
| KrAuO | −9.81 | −25.0 (51.4) | −23.4 (48.1) | 38.8 | −0.25 (0.5) |
| XeAuO | −14.83 | −37.8 (53.4) | −32.7 (46.2) | 56.0 | −0.28 (0.4) |
| RnAuO | −16.58 | −40.1 (53.8) | −34.1 (45.8) | 57.8 | −0.35 (0.5) |

**Table S2**. EDA results of the NgMCN (M = Cu, Ag, Au) clusters with Ng and MCN as fragments calculated at the PBE-D3/QZ4P//CCSD(T)/def2-TZVPPD level. All energy terms are in kcal/mol. (The percentage values within the parentheses show the contribution toward the total attractive interaction, $\Delta E_{elstat}$ + $\Delta E_{orb}$ + $\Delta E_{disp}$). [Reprinted from Ref. [75] with permission from John Wiley and Sons. © 2015 Wiley Periodicals, Inc.]

| Systems | $\Delta E_{int}$ | $\Delta E_{elstat}$ | $\Delta E_{orb}$ | $\Delta E_{Pauli}$ | $\Delta E_{disp}$ |
|---|---|---|---|---|---|
| ArCuCN | −8.5 | −11.6 (45.5) | −13.7 (53.7) | 16.9 | −0.2 (0.8) |
| KrCuCN | −11.0 | −15.0 (47.0) | −16.7 (52.4) | 20.9 | −0.2 (0.6) |
| XeCuCN | −15.5 | −18.7 (45.9) | −21.7 (53.3) | 25.2 | −0.3 (0.7) |
| RnCuCN | −16.6 | −19.9 (46.2) | −22.9 (53.1) | 26.6 | −0.3 (0.7) |
| ArAgCN | −5.2 | −7.0 (47.3) | −7.5 (50.7) | 9.7 | −0.3 (2.0) |
| KrAgCN | −7.5 | −11.2 (49.8) | −10.9 (48.4) | 15.0 | −0.4 (1.8) |
| XeAgCN | −11.0 | −17.1 (52.0) | −15.4 (46.8) | 21.9 | −0.4 (1.2) |
| RnAgCN | −12.4 | −19.7 (52.8) | −17.1 (45.8) | 24.9 | −0.5 (1.3) |
| ArAuCN | −7.9 | −16.1 (48.6) | −16.7 (50.5) | 25.2 | −0.3 (0.9) |
| KrAuCN | −11.1 | −22.4 (50.7) | −21.5 (48.6) | 33.2 | −0.3 (0.7) |
| XeAuCN | −16.1 | −32.3 (52.5) | −28.8 (46.8) | 45.4 | −0.4 (0.7) |
| RnAuCN | −17.8 | −35.2 (53.1) | −30.6 (46.2) | 48.5 | −0.5 (0.8) |

**Table S3**. Electron density descriptors (au) at the bond critical points (BCPs) in between Ng and M atoms in NgMCN obtained from the wave functions generated at the MP2/def2-TZVPPD/WTBS//CCSD(T)/def2-TZVPPD level (All electron WTBS basis set is used only for Ag, Au, Xe, and Rn). [Reprinted from Ref. [75] with permission from John Wiley and Sons. © 2015 Wiley Periodicals, Inc.]

| Systems | BCP | $\rho(r_c)$ | $\nabla^2\rho(r_c)$ | $G(r_c)$ | $V(r_c)$ | $H(r_c)$ | $G(r_c)/\rho(r_c)$ |
|---|---|---|---|---|---|---|---|
| ArCuCN | Ar-Cu | 0.046 | 0.229 | 0.063 | −0.07 | −0.007 | 1.37 |
| KrCuCN | Kr-Cu | 0.049 | 0.198 | 0.058 | −0.067 | −0.009 | 1.184 |
| XeCuCN | Xe-Cu | 0.052 | 0.132 | 0.047 | −0.061 | −0.014 | 0.904 |
| RnCuCN | Rn-Cu | 0.053 | 0.109 | 0.042 | −0.057 | −0.015 | 0.792 |
| ArAgCN | Ar-Ag | 0.028 | 0.141 | 0.034 | −0.033 | 0.001 | 1.214 |
| KrAgCN | Kr-Ag | 0.033 | 0.146 | 0.038 | −0.039 | −0.001 | 1.152 |
| XeAgCN | Xe-Ag | 0.034 | 0.138 | 0.036 | −0.037 | −0.001 | 1.059 |
| RnAgCN | Rn-Ag | 0.036 | 0.13 | 0.035 | −0.038 | −0.003 | 0.972 |
| ArAuCN | Ar-Au | 0.046 | 0.244 | 0.062 | −0.063 | −0.001 | 1.348 |
| KrAuCN | Kr-Au | 0.05 | 0.222 | 0.06 | −0.065 | −0.005 | 1.2 |
| XeAuCN | Xe-Au | 0.046 | 0.195 | 0.052 | −0.056 | −0.004 | 1.13 |
| RnAuCN | Rn-Au | 0.046 | 0.175 | 0.048 | −0.053 | −0.005 | 1.043 |

**Table S4**. Electron density descriptors (au) at the BCPs in between Ng and M centers obtained from the wave functions generated at the MP2/cc-pVTZ/WTBS//CCSD(T)/VTZ level (All electron WTBS basis set is used only for Ag,

Au, Xe, and Rn). [Reprinted from Ref. [76] with permission from John Wiley and Sons. © 2015 Wiley Periodicals, Inc.]

| **Systems** | **BCP** | $\rho(r_c)$ | $\nabla^2\rho(r_c)$ | $G(r_c)$ | $V(r_c)$ | $H(r_c)$ | $G(r_c)/\rho(r_c)$ |
|---|---|---|---|---|---|---|---|
| ArCuO | Ar-Cu | 0.045 | 0.271 | 0.071 | 20.075 | 20.004 | 1.578 |
| KrCuO | Kr-Cu | 0.045 | 0.271 | 0.071 | 20.075 | 20.004 | 1.578 |
| XeCuO | Xe-Cu | 0.043 | 0.188 | 0.052 | 20.057 | 20.005 | 1.209 |
| RnCuO | Rn-Cu | 0.041 | 0.161 | 0.046 | 20.051 | 20.005 | 1.122 |
| ArAgO | Ar-Ag | 0.028 | 0.147 | 0.035 | 20.034 | 0.001 | 1.250 |
| KrAgO | Kr-Ag | 0.031 | 0.153 | 0.038 | 20.037 | 0.001 | 1.226 |
| XeAgO | Xe-Ag | 0.037 | 0.154 | 0.041 | 20.043 | 20.002 | 1.108 |
| RnAgO | Rn-Ag | 0.037 | 0.141 | 0.038 | 20.041 | 20.003 | 1.027 |
| ArAuO | Ar-Au | 0.042 | 0.236 | 0.059 | 20.059 | 0.000 | 1.405 |
| KrAuO | Kr-Au | 0.042 | 0.232 | 0.058 | 20.057 | 0.001 | 1.381 |
| XeAuO | Xe-Au | 0.047 | 0.210 | 0.055 | 20.059 | 20.004 | 1.170 |
| RnAuO | Rn-Au | 0.046 | 0.186 | 0.050 | 20.054 | 20.004 | 1.087 |

**Table S5**. Different topological descriptors (au) at the bond critical points (BCP) in between Ng and M atoms in NgMY and $Ng_2M_2Y$ (M = Cu, Ag; Y = $NO_3$, $SO_4$, $CO_3$) complexes at the MPW1B95/def2-TZVP level. [Reprinted from Ref. [77] with permission from Springer Nature. © 2016, Indian Academy of Sciences]

| **Systems** | **BCP** | $\rho(r_c)$ | $\nabla^2\rho(r_c)$ | $G(r_c)$ | $V(r_c)$ | $H(r_c)$ | $G(r_c)/\rho(r_c)$ |
|---|---|---|---|---|---|---|---|
| $ArCuNO_3$ | Ar-Cu | 0.046 | 0.231 | 0.062 | −0.067 | −0.004 | 1.348 |
| $KrCuNO_3$ | Kr-Cu | 0.05 | 0.209 | 0.059 | −0.066 | −0.007 | 1.180 |
| $XeCuNO_3$ | Xe-Cu | 0.052 | 0.173 | 0.053 | −0.062 | −0.009 | 1.019 |
| $RnCuNO_3$ | Rn-Cu | 0.05 | 0.150 | 0.046 | −0.055 | −0.009 | 0.920 |
| $ArAgNO_3$ | Ar-Ag | 0.028 | 0.122 | 0.031 | −0.031 | 0.000 | 1.107 |
| $KrAgNO_3$ | Kr-Ag | 0.036 | 0.129 | 0.035 | −0.038 | −0.003 | 0.972 |
| $XeAgNO_3$ | Xe-Ag | 0.043 | 0.128 | 0.038 | −0.043 | −0.006 | 0.884 |
| $RnAgNO_3$ | Rn-Ag | 0.043 | 0.115 | 0.035 | −0.042 | −0.006 | 0.814 |
| $ArCuSO_4$ | Ar-Cu | 0.045 | 0.227 | 0.061 | −0.065 | −0.004 | 1.356 |
| $KrCuSO_4$ | Kr-Cu | 0.05 | 0.206 | 0.059 | −0.066 | −0.007 | 1.180 |
| $XeCuSO_4$ | Xe-Cu | 0.053 | 0.171 | 0.052 | −0.062 | −0.009 | 0.981 |
| $RnCuSO_4$ | Rn-Cu | 0.05 | 0.149 | 0.046 | −0.055 | −0.009 | 0.920 |
| $ArAgSO_4$ | Ar-Ag | 0.028 | 0.121 | 0.03 | −0.031 | 0.000 | 1.071 |
| $KrAgSO_4$ | Kr-Ag | 0.036 | 0.129 | 0.035 | −0.038 | −0.003 | 0.972 |

| | | | | | | | |
|---|---|---|---|---|---|---|---|
| $XeAgSO_4$ | Xe-Ag | 0.043 | 0.126 | 0.037 | −0.043 | −0.006 | 0.860 |
| $RnAgSO_4$ | Rn-Ag | 0.043 | 0.113 | 0.035 | −0.041 | −0.007 | 0.814 |
| $ArCuCO_3$ | Ar-Cu | 0.039 | 0.188 | 0.049 | −0.051 | −0.002 | 1.256 |
| $KrCuCO_3$ | Kr-Cu | 0.043 | 0.17 | 0.047 | −0.051 | −0.005 | 1.093 |
| $XeCuCO_3$ | Xe-Cu | 0.046 | 0.144 | 0.043 | −0.049 | −0.007 | 0.935 |
| $RnCuCO_3$ | Rn-Cu | 0.044 | 0.125 | 0.038 | −0.044 | −0.007 | 0.864 |
| $Ar_2Cu_2SO_4$ | Ar-Cu | 0.044 | 0.221 | 0.059 | −0.062 | −0.004 | 1.341 |
| $Kr_2Cu_2SO_4$ | Kr-Cu | 0.049 | 0.205 | 0.058 | −0.064 | −0.007 | 1.184 |
| $Xe_2Cu_2SO_4$ | Xe-Cu | 0.052 | 0.172 | 0.052 | −0.061 | −0.009 | 1.000 |
| $Rn_2Cu_2SO_4$ | Rn-Cu | 0.049 | 0.149 | 0.046 | −0.054 | −0.008 | 0.939 |
| $Ar_2Ag_2SO_4$ | Ar-Ag | 0.026 | 0.114 | 0.028 | −0.028 | 0.000 | 1.077 |
| $Kr_2Ag_2SO_4$ | Kr-Ag | 0.034 | 0.125 | 0.034 | −0.037 | −0.003 | 1.000 |
| $Xe_2Ag_2SO_4$ | Xe-Ag | 0.042 | 0.126 | 0.037 | −0.042 | −0.005 | 0.881 |
| $Rn_2Ag_2SO_4$ | Rn-Ag | 0.042 | 0.113 | 0.034 | −0.041 | −0.006 | 0.810 |
| $Ar_2Au_2SO_4$ | Ar-Au | 0.058 | 0.242 | 0.069 | −0.078 | −0.009 | 1.190 |
| $Kr_2Au_2SO_4$ | Kr-Au | 0.066 | 0.216 | 0.067 | −0.080 | −0.013 | 1.015 |
| $Xe_2Au_2SO_4$ | Xe-Au | 0.071 | 0.166 | 0.06 | −0.078 | −0.018 | 0.845 |
| $Rn_2Au_2SO_4$ | Rn-Au | 0.068 | 0.143 | 0.054 | −0.072 | −0.018 | 0.794 |
| $Ar_2Ag_2CO_3$ | Ar-Ag | 0.026 | 0.111 | 0.028 | −0.027 | 0.000 | 1.077 |
| $Kr_2Ag_2CO_3$ | Kr-Ag | 0.034 | 0.124 | 0.033 | −0.036 | −0.003 | 0.971 |
| $Xe_2Ag_2CO_3$ | Xe-Ag | 0.042 | 0.125 | 0.037 | −0.042 | −0.005 | 0.881 |
| $Rn_2Ag_2CO_3$ | Rn-Ag | 0.041 | 0.112 | 0.034 | −0.040 | −0.006 | 0.829 |
| $Ar_2Au_2CO_3$ | Ar-Au | 0.055 | 0.234 | 0.066 | −0.073 | −0.007 | 1.200 |
| $Kr_2Au_2CO_3$ | Kr-Au | 0.063 | 0.212 | 0.065 | −0.077 | −0.012 | 1.032 |
| $Xe_2Au_2CO_3$ | Xe-Au | 0.069 | 0.166 | 0.058 | −0.075 | −0.017 | 0.841 |
| $Rn_2Au_2CO_3$ | Rn-Au | 0.066 | 0.143 | 0.053 | −0.070 | −0.017 | 0.803 |

**Table S6**. Topological descriptors (au) at the line critical point between Ng and M atoms in $[NgM\text{-}(bipy)]^+$ obtained from wave function generated at the MPW1B95/cc-pVTZ/WTBS//MPW1B95/cc-pVTZ level (All electron WTBS basis set is used only for Cu, Ag, Au, Kr, Xe and Rn). [Reprinted from Ref. [85] with permission from John Wiley and Sons. © 2016 Wiley-VCH Verlag GmbH & Co. KGaA, Weinheim]

| Systems | $\rho(r_c)$ | $\nabla^2\rho(r_c)$ | $G(r_c)$ | $V(r_c)$ | $H(r_c)$ | $G(r_c)/\rho(r_c)$ |
|---|---|---|---|---|---|---|
| $[ArCu\text{-}(bipy)]^+$ | 0.042 | 0.211 | 0.060 | −0.063 | −0.003 | 1.417 |
| $[KrCu\text{-}(bipy)]^+$ | 0.042 | 0.208 | 0.055 | −0.059 | −0.003 | 1.331 |
| $[XeCu\text{-}(bipy)]^+$ | 0.044 | 0.177 | 0.050 | −0.056 | −0.006 | 1.146 |

| | | | | | | |
|---|---|---|---|---|---|---|
| [RnCu-(bipy)]+ | 0.042 | 0.151 | 0.044 | −0.050 | −0.006 | 1.045 |
| [ArAg-(bipy)]+ | 0.029 | 0.143 | 0.035 | −0.034 | 0.001 | 1.211 |
| [KrAg-(bipy)]+ | 0.036 | 0.15 | 0.039 | −0.041 | −0.002 | 1.097 |
| [XeAg-(bipy)]+ | 0.038 | 0.151 | 0.040 | −0.043 | −0.003 | 1.061 |
| [RnAg-(bipy)]+ | 0.038 | 0.136 | 0.037 | −0.040 | −0.003 | 0.984 |
| [ArAu-(bipy)]+ | 0.045 | 0.224 | 0.057 | −0.059 | −0.001 | 1.276 |
| [KrAu-(bipy)]+ | 0.048 | 0.246 | 0.063 | −0.065 | −0.002 | 1.312 |
| [XeAu-(bipy)]+ | 0.052 | 0.215 | 0.059 | −0.065 | −0.006 | 1.135 |
| [RnAu-(bipy)]+ | 0.051 | 0.186 | 0.053 | −0.059 | −0.006 | 1.044 |

**Table S7**. Energy decomposition analysis (EDA) results of the [NgM-(bipy)]+ complexes taking Ng as one fragment and [M-(bipy)]+ as another, studied at the BLYP-D3(BJ)/QZ4P//MPW1B95/cc-pVTZ level. All the energy terms are in kcal/mol. The percentage values within the parentheses show the contribution towards the total attractive interaction, $\Delta E_{elstat} + \Delta E_{orb} + \Delta E_{disp}$. [Reprinted from Ref. [85] with permission from John Wiley and Sons. © 2016 Wiley-VCH Verlag GmbH & Co. KGaA, Weinheim]

| Systems | $\Delta E_{int}$ | $\Delta E_{elstat}$ | $\Delta E_{orb}$ | $\Delta E_{Pauli}$ | $\Delta E_{disp}$ |
|---|---|---|---|---|---|
| [ArCu-(bipy)]+ | −8.5 | −15.8 (48.1%) | −14.8 (45.1%) | 24.3 | −2.2 (6.8%) |
| [KrCu-(bipy)]+ | −11.1 | −21.1 (50.1%) | −18.2 (43.2%) | 30.9 | −2.8 (6.7%) |
| [XeCu-(bipy)]+ | −17.1 | −28.0 (49.9%) | −24.4 (43.5%) | 38.9 | −3.7 (6.7%) |
| [RnCu-(bipy)]+ | −18.7 | −27.6 (49.0%) | −24.8 (43.9%) | 37.7 | −4.0 (7.1%) |
| [ArAg-(bipy)]+ | −5.7 | −8.8 (45.7%) | −8.5 (43.9%) | 13.6 | −2.0 (10.5%) |
| [KrAg-(bipy)]+ | −8.5 | −14.2 (48.7%) | −12.4 (42.6%) | 20.6 | −2.5 (8.7%) |
| [XeAg-(bipy)]+ | −13.0 | −23.6 (51.8%) | −18.7 (40.9%) | 32.6 | −3.3 (7.3%) |
| [RnAg-(bipy)]+ | −15.0 | −25.0 (51.5%) | −20.0 (41.2%) | 33.5 | −3.5 (7.3%) |
| [ArAu-(bipy)]+ | −8.0 | −19.3 (50.1%) | −17.0 (44.0%) | 30.5 | −2.3 (5.8%) |
| [KrAu-(bipy)]+ | −13.2 | −31.4 (52.1%) | −26.0 (43.2%) | 47 | −2.9 (4.8%) |
| [XeAu-(bipy)]+ | −20.3 | −46.9 (54.4%) | −35.6 (41.3%) | 65.9 | −3.7 (4.3%) |

| | | | | | |
|---|---|---|---|---|---|
| $[RnAu\text{-}(bipy)]^+$ | −22.9 | −48.5 (54.4%) | −36.7 (41.2%) | 66.3 | −3.9 (4.4%) |

**Table S8**. EDA results of MNgCCH and NgMCCH at the PBE-D3(BJ)/QZ4P//CCSD(T)/cc-pVTZ level. All energy values are in kcal/mol. The values in parentheses are percentage contribution toward the total attraction, $\Delta E_{elstat} + \Delta E_{orb} + \Delta E_{disp}$. [Reprinted from Ref. [86] with permission from American Chemical Society. © 2017, American Chemical Society]

| Systems | Fragments | $\Delta E_{int}$ | $\Delta E_{elstat}$ | $\Delta E_{orb}$ | $\Delta E_{Pauli}$ | $\Delta E_{disp}$ |
|---|---|---|---|---|---|---|
| CuXeCCH | Cu + XeCCH | −33.3 | −58.6 (52.9) | −51.3 (46.3) | 77.5 | −0.9 (0.8) |
| CuRnCCH | Cu + RnCCH | −38.1 | −64.1 (54.9) | −51.8 (44.3) | 78.7 | −0.9 (0.7) |
| AgXeCCH | Ag + XeCCH | −25.8 | −40.0 (50.5) | −38.2 (48.2) | 53.3 | −1.0 (1.3) |
| AgRnCCH | Ag + RnCCH | −31.1 | −50.4 (54.0) | −41.9 (44.9) | 62.2 | −1.0 (1.1) |
| AuXeCCH | Au + XeCCH | −38.5 | −75.9 (55.2) | −60.6 (44.0) | 99.2 | −1.1 (0.8) |
| AuRnCCH | Au + RnCCH | −45.1 | −84.6 (57.4) | −61.8 (41.9) | 102.4 | −1.1 (0.7) |
| ArCuCCH | Ar + CuCCH | −8.9 | −12.3 (45.5) | −14.0 (51.9) | 18.1 | −0.7 (2.6) |
| KrCuCCH | Kr + CuCCH | −11.2 | −15.8 (47.1) | −17.0 (50.5) | 22.4 | −0.8 (2.4) |
| XeCuCCH | Xe + CuCCH | −14.9 | −19.9 (47.4) | −21.1 (50.2) | 27.2 | −1.0 (2.4) |
| RnCuCCH | Rn + CuCCH | −15.7 | −20.4 (47.3) | −21.7 (50.2) | 27.5 | −1.0 (2.4) |
| ArAgCCH | Ar + AgCCH | −5.3 | −7.2 (47.6) | −7.2 (47.6) | 9.9 | −0.7 (4.8) |
| KrAgCCH | Kr + AgCCH | −7.5 | −11.6 (50.7) | −10.4 (45.5) | 15.5 | −0.9 (3.8) |
| XeAgCCH | Xe + AgCCH | −10.9 | −17.9 (53.0) | −14.8 (43.8) | 22.9 | −1.1 (3.1) |
| RnAgCCH | Rn + AgCCH | −12.1 | −19.6 (53.6) | −15.8 (43.4) | 24.5 | −1.1 (3.0) |
| ArAuCCH | Ar + AuCCH | −7.6 | −13.3 (47.6) | −13.8 (49.5) | 20.3 | −0.8 (2.9) |
| KrAuCCH | Kr + AuCCH | −10.6 | −20.0 (50.3) | −18.8 (47.4) | 29.1 | −0.9 (2.3) |

| | | | | | | |
|---|---|---|---|---|---|---|
| XeAuCCH | Xe + AuCCH | −15.4 | −29.8 (52.5) | −25.9 (45.5) | 41.5 | −1.1 (2.0) |
| RnAuCCH | Rn + AuCCH | −16.9 | −32.3 (53.2) | −27.3 (44.9) | 43.9 | −1.2 (1.9) |

**Table S9**. Electron density descriptors (au) at the BCPs of M-Ng and Ng-C bonds in MNgCCH compounds obtained from the wave functions generated at the MP2/ccpVTZ/WTBS//CCSD(T)/cc-pVTZ level (WTBS for Cu, Ag, Au, Xe and Rn atoms). [Reprinted from Ref. [86] with permission from American Chemical Society. © 2017, American Chemical Society]

| Systems | BCP | $\rho(r_c)$ | $\nabla^2\rho(r_c)$ | $G(r_c)$ | $V(r_c)$ | $H(r_c)$ | $G(r_c)/\rho(r_c)$ |
|---|---|---|---|---|---|---|---|
| CuXeCCH | Cu–•–Xe | 0.048 | 0.155 | 0.047 | −0.1 | −0.008 | 0.979 |
| CuRnCCH | Cu–•–Rn | 0.047 | 0.134 | 0.042 | −0.1 | −0.009 | 0.894 |
| AgXeCCH | Ag–•–Xe | 0.038 | 0.123 | 0.034 | 0.0 | −0.003 | 0.895 |
| AgRnCCH | Ag–•–Rn | 0.040 | 0.119 | 0.034 | 0.0 | −0.005 | 0.850 |
| AuXeCCH | Au–•–Xe | 0.055 | 0.178 | 0.052 | −0.1 | −0.008 | 0.945 |
| AuRnCCH | Au–•–Rn | 0.054 | 0.153 | 0.047 | −0.1 | −0.009 | 0.870 |

**Table S10**. Electron density descriptors (in au) calculated at the MP2/cc-pVTZ/WTBS//CCSD(T)/VTZ level. [Reprinted from Ref. [87] with permission from American Chemical Society. © 2018, American Chemical Society]

| Systems | BCP | $\rho(r_c)$ | $\nabla^2\rho(r_c)$ | $G(r_c)$ | $V(r_c)$ | $H(r_c)$ | $G(r_c)/\rho(r_c)$ |
|---|---|---|---|---|---|---|---|
| CuCCKrH | Kr–C | 0.080 | 0.104 | 0.050 | −0.073 | −0.024 | 0.621 |
| CuCCXeH | Xe–C | 0.082 | 0.084 | 0.051 | −0.081 | −0.030 | 0.626 |
| CuCCRnH | Rn–C | 0.078 | 0.097 | 0.053 | −0.081 | −0.028 | 0.676 |
| AgCCKrH | Kr–C | 0.080 | 0.103 | 0.050 | −0.073 | −0.024 | 0.619 |
| AgCCXeH | Xe–C | 0.082 | 0.083 | 0.051 | −0.081 | −0.030 | 0.622 |
| AgCCRnH | Rn–C | 0.082 | 0.083 | 0.051 | −0.081 | −0.030 | 0.622 |
| AuCCKrH | Kr–C | 0.079 | 0.106 | 0.050 | −0.073 | −0.023 | 0.628 |
| AuCCXeH | Xe–C | 0.079 | 0.085 | 0.050 | −0.078 | −0.028 | 0.626 |
| AuCCRnH | Rn–C | 0.076 | 0.096 | 0.051 | −0.078 | −0.027 | 0.673 |
| CuCCKrH | Kr–H | 0.118 | −0.040 | 0.060 | −0.130 | −0.070 | 0.118 |
| CuCCXeH | Xe–H | 0.110 | 0.021 | 0.067 | −0.128 | −0.062 | 0.110 |
| CuCCRnH | Rn–H | 0.100 | 0.079 | 0.070 | −0.120 | −0.050 | 0.100 |

| | | | | | | | |
|---|---|---|---|---|---|---|---|
| AgCCKrH | Kr–H | 0.116 | −0.034 | 0.060 | −0.128 | −0.068 | 0.116 |
| AgCCXeH | Xe–H | 0.109 | 0.022 | 0.066 | −0.127 | −0.060 | 0.109 |
| AgCCRnH | Rn–H | 0.099 | 0.079 | 0.069 | −0.119 | −0.050 | 0.099 |
| AuCCKrH | Kr–H | 0.130 | −0.075 | 0.067 | −0.153 | −0.086 | 0.130 |
| AuCCXeH | Xe–H | 0.115 | 0.020 | 0.072 | −0.140 | −0.067 | 0.115 |
| AuCCRnH | Rn–H | 0.104 | 0.085 | 0.075 | −0.130 | −0.054 | 0.104 |

**Table S11**. EDA Results of MCCNgH at the PBE-D3/QZ4P//CCSD(T)/VTZ Level. All energy values are in kcal/mol. The values in parentheses are the percentage contribution toward the total attraction, $\Delta E_{elstat}$ + $\Delta E_{orb}$ + $\Delta E_{disp}$. [Reprinted from Ref. [87] with permission from American Chemical Society. © 2018, American Chemical Society]

| **Systems** | **Fragments** | **$\Delta E_{int}$** | **$\Delta E_{Pauli}$** | **$\Delta E_{elstat}$** | **$\Delta E_{orb}$** | **$\Delta E_{disp}$** |
|---|---|---|---|---|---|---|
| CuCCKrH | CuCCKr + H | −27.1 | 94.6 | −50.3 (41.3) | −71.3 (58.6) | −0.1 (0.1) |
| CuCCXeH | CuCCXe + H | −40.3 | 96.0 | −56.3 (41.3) | −80.0 (58.7) | −0.1 (0.1) |
| CuCCRnH | CuCCRn + H | −44.3 | 86.3 | −55.3 (42.3) | −75.2 (57.6) | −0.1 (0.1) |
| AgCCKrH | AgCCKr + H | −27.5 | 91.2 | −49.0 (41.2) | −69.7 (58.7) | −0.1 (0.1) |
| AgCCXeH | AgCCXe + H | −39.9 | 95.4 | −55.8 (41.2) | −79.4 (58.7) | −0.1 (0.1) |
| AgCCRnH | AgCCRn + H | −43.9 | 86.1 | −55.0 (42.3) | −74.9 (57.6) | −0.1 (0.1) |
| AuCCKrH | AuCCKr + H | −29.5 | 104.5 | −54.8 (40.9) | −79.0 (59.0) | −0.1 (0.1) |
| AuCCXeH | AuCCXe + H | −40.6 | 101.6 | −58.6 (41.2) | −83.5 (58.7) | −0.1 (0.1) |
| AuCCRnH | AuCCRn + H | −44.6 | 90.4 | −57.3 (42.4) | −77.7 (57.5) | −0.1 (0.1) |
| CuCCKrH | $CuCC^-$ + $KrH^+$ | −164.2 | 120.0 | −175.7 (61.8) | −108.1 (38.0) | −0.5 (0.2) |
| CuCCXeH | $CuCC^-$ + $XeH^+$ | −162.4 | 144.2 | −195.6 (63.8) | −110.4 (36.0) | −0.5 (0.2) |
| CuCCRnH | $CuCC^-$ + $RnH^+$ | −163.8 | 139.5 | −201.1 (66.3) | −101.6 (33.5) | −0.6 (0.2) |
| AgCCKrH | $AgCC^-$ + $KrH^+$ | −165.4 | 114.0 | −170.9 (61.2) | −108.0 (38.7) | −0.4 (0.1) |
| AgCCXeH | $AgCC^-$ + $XeH^+$ | −163.2 | 142.5 | −192.7 (63.0) | −112.5 (36.8) | −0.5 (0.2) |
| AgCCRnH | $AgCC^-$ + $RnH^+$ | −164.6 | 137.6 | −197.9 (65.5) | −103.7 (34.3) | −0.6 (0.2) |
| AuCCKrH | $AuCC^-$ + $KrH^+$ | −154.0 | 109.9 | −166.2 (63.0) | −97.3 (36.9) | −0.5 (0.2) |
| AuCCXeH | $AuCC^-$ + $XeH^+$ | −153.8 | 132.8 | −183.8 (64.2) | −102.1 (35.6) | −0.6 (0.2) |
| AuCCRnH | $AuCC^-$ + $RnH^+$ | −155.5 | 129.0 | −189.2 (66.5) | −94.7 (33.3) | −0.7 (0.2) |
